# A 1-Pot Synthesis of the SARS-CoV-2 M^pro^ Inhibitor Nirmatrelvir, the Key Ingredient in Paxlovid

**DOI:** 10.1021/acs.orglett.2c03683

**Published:** 2022-12-07

**Authors:** Juan C. Caravez, Karthik S. Iyer, Rahul D. Kavthe, Joseph R. A. Kincaid, Bruce H. Lipshutz

**Affiliations:** Department of Chemistry & Biochemistry, University of California, Santa Barbara, Santa Barbara, California 93106, United States

## Abstract

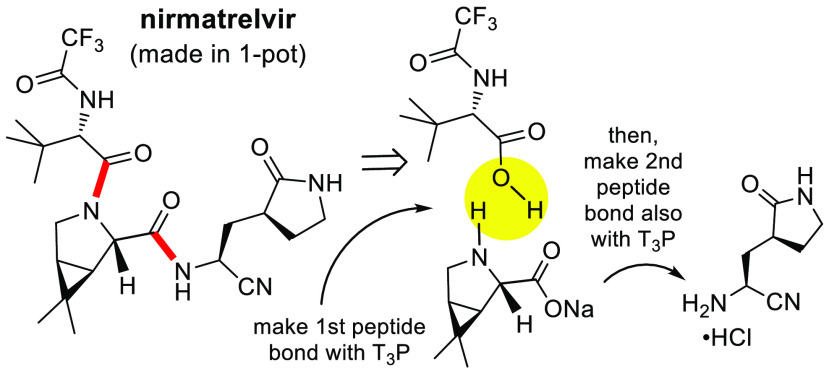

A newly devised route to the Pfizer drug nirmatrelvir is reported that reduces the overall sequence to a 1-pot process and relies on a commercially available, green coupling reagent, T3P. The overall yield of the targeted material, isolated as its MTBE solvate, is 64%.

While impressive progress resulting in millions of lives being saved has been made by the pharmaceutical industry by providing highly effective vaccines against the continuing worldwide pandemic,^1^ rates of serious infection and deaths caused by COVID-19 continue to plague society.^2^ Newly developed treatments involving antivirals have recently been introduced that add significantly to the toolbox of drugs currently available, which include Merck’s molnupiravir^3^ and Pfizer’s nirmatrelvir, the major component found in Paxlovid.^4^ The latter was approved for emergency use by the U.S. Food and Drug Administration in late 2021^4^ and has been enthusiastically distributed by the medical profession as an effective treatment, being projected to bring in revenues on the order of 22 billion dollars in 2022 alone.^5^ The route being used by Pfizer and its collaborators has evolved from the initial disclosure in 2021, with improvements leading to a 5-step commercial process that affords nirmatrelvir in 62% overall yield.^4,6^ The sequence features reductions not only in step count (from 7 to 5), but also noted are several “green” metrics, such as PMI/step (21), cumulative PMI (108), and energy usage (1513 MJ). Several other laboratories have also described very recent synthetic efforts toward further improving the route to nirmatrelvir, looking to lower the overall cost associated with making this important target. For example, Nuckols and Shanahan et al. have described a route bearing an overall yield of 48%.^7^ Likewise, Ruijter and Turner et al. have also contributed, using a chemoenzymatic approach.^8^

Our interest in developing a process that is both cost-effective in terms of potential access by the third world, and environmentally responsible led us to recently disclose a 3-pot, 7-step sequence^9^ that focuses on facile formation of 2-mercaptopyridine-derived thioester intermediates^10a,10b^ that can then be converted to the targeted peptides. This technology shows considerable promise as an alternative to traditional peptide coupling reagents, since it converts carboxylic acids to amides/peptides under very green conditions; e.g., either neat, in highly concentrated EtOAc, or in aqueous micellar media.^11^ These thioesters, with or more likely, without isolation, are subject to introduction of the amine leading to the desired amide/peptide. Applying this approach, we prepared nirmatrelvir in 70% overall isolated yield.^9^

Notwithstanding this synthesis, which remains as of this writing the most effective to date, the need for 7 steps which includes a protecting group removal (i.e., the *N*-Boc group from the starting *N*-Boc-*t*-leucine) provided the incentive to further streamline the route to nirmatrelvir. Moreover, the sequence as originally developed was hampered by our inability to make the corresponding thioester using the alternative educt, the trifluoroacetamide derivative of *t*-leucine, which would avoid both *N*-Boc deprotection and eventual insertion of the required trifluoroacetamide, thereby shortening the route by two steps. In this report we describe the successful search for, and reduction to practice of, a 1-pot synthesis (Figure 1) using an alternative, green coupling technology that affords the targeted drug as its MTBE solvate^4^ in 64% overall isolated yield.

**Figure 1:**
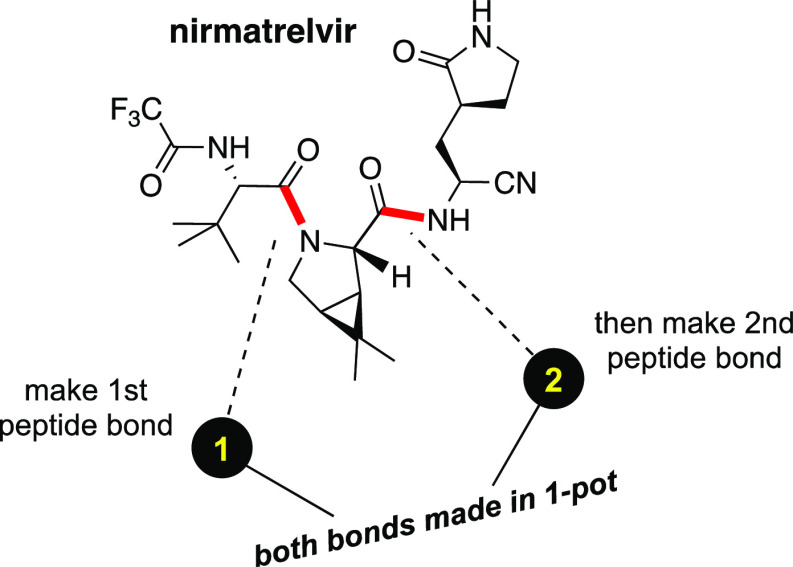
Strategy associated with a 1-pot synthesis of nirmatrelvir.

## Results and Discussion

Because of its low cost, availability, and extended use on scale as an activating agent for carboxylic acids,^12^ thionyl chloride was originally screened in anticipation that the resulting acid chloride would facilitate coupling of **1** with the bicyclic proline (as its lithium salt, **2b**, see the Supporting Information (SI), Table S1) to arrive at intermediate acid **3** following an acidic aqueous work up and precipitation. Unfortunately, after several attempts at optimization, the yields of **3** were consistently low (see SI, Table S1) and several impurities could be detected by ^1^H NMR. Aside from the difficulty associated with handling this highly activated acid chloride due to its sensitivity to moisture, especially on a smaller, academic scale, the potential for the acid chloride to cyclize to form an oxazolone intermediate was also appreciated.^12,13^ Moreover, the possibility that residual M–OH (M = Li or Na) was present from hydrolysis of the precursor methyl ester en route to the bicyclic proline salt further encouraged investigation into an alternative strategy. Consideration of the numerous peptide coupling reagents in terms of both cost and “greenness” turned our focus to alkyl chloroformates, which have been widely used on scale for this purpose. They are known for minimizing racemization, while imparting far greater stability to the resulting intermediates, especially to adventitious moisture.^12,14,15^ The use of ethyl chloroformate (ECF), along with equimolar quantities of base (*N*-methylmorpholine, NMM, or Hunig’s base, DIPEA), did not provide the desired high yields (Table 1, entries 1 and 2), although the overall cleanliness of the reaction and isolated product did improve relative to the acid chloride approach (see SI, Table S2 for more information). The lower yields obtained were not due to incomplete conversion; rather, the issue stemmed from the regioselectivity of attack by the proline nitrogen on this unhindered alkyl chloroformate.^14^ In efforts to improve the regioselectivity by using the more sterically hindered, commercially available 2-ethylhexyl chloroformate, the yield, in fact, decreased by about 50%, forcing evaluation of additional coupling reagents for generating the first peptide bond (entry 4). Several coupling reagents were screened (see SI, Table S2) leading to varying results. For example, pivaloyl chloride resulted in pivalic acid impurities following acidic workup. Both MsCl and TsCl, used by Pfizer,^6^ aside from giving only modest yields, were deemed less attractive from both an environmental and human health perspective.^16^ Eventually, it was found that propane phosphonic acid anhydride (T3P) showed considerable potential as a coupling reagent^17^ that appeared to offer several attractive features (i.e., efficiency, lack of stereochemical issues in terms of diastereomer formation, environmental concerns, economics, etc.), and thus, was subjected to intense evaluation (Table 1, entries 5–9).

**Scheme 1:**
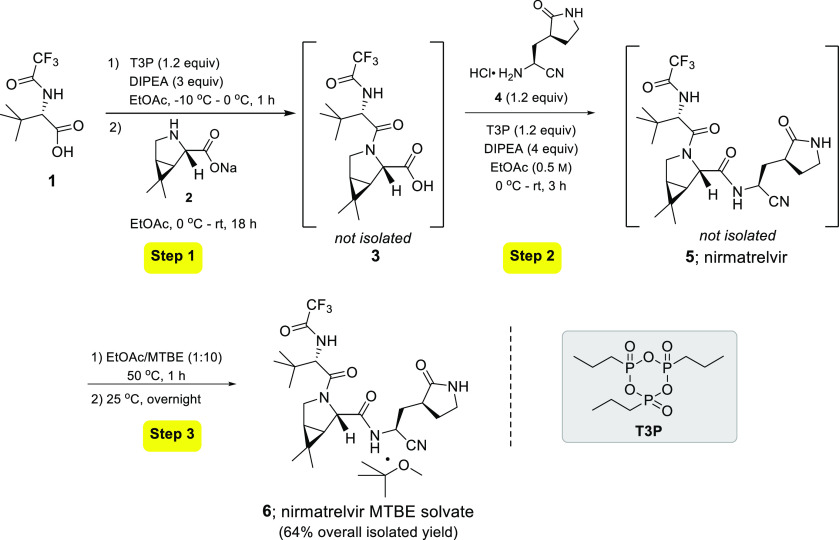
A 1-Pot, 3-Step Synthesis of Nirmatrelvir MTBE Solvate (**6**)

**Table 1:**
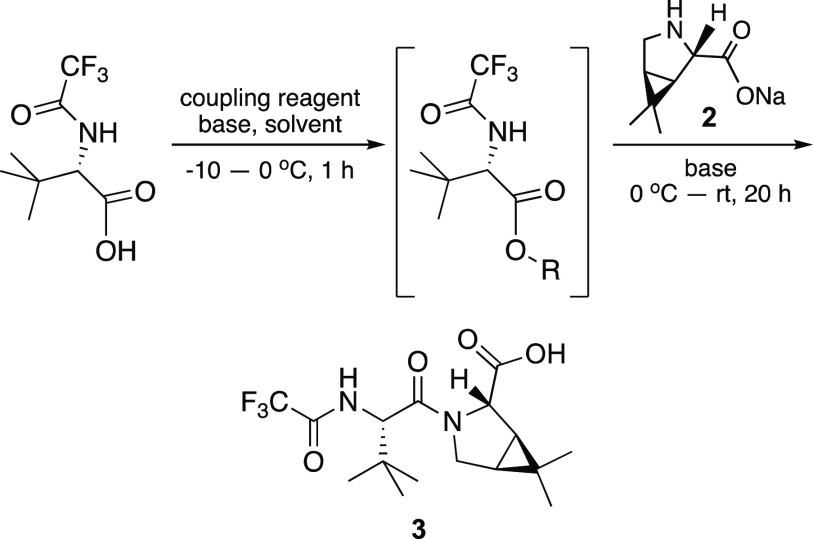
Screening of Peptide Coupling Reagents

entry^a^	coupling reagent (equiv)	amine (2) (equiv)	solvent	concentration (M)	base (equiv)	yield (%)^b^
1	ECF (1)	0.83	EtOAc	0.5	NMM (1)	40
2	ECF (1.2)	1.2	THF	0.5	NMM (1)	45
3	ECF (1.2)	1.2	2-MeTHF	0.5	NMM (1.2)	60
4	2-EHCF (1.2)	1.2	2-MeTHF	0.5	NMM (1.2)	25
5	T3P (1.2)	1.2	EtOAc	0.3	DIPEA (3)	73
6^c^	T3P (1.2)	1.2	EtOAc	1.4–0.8	DIPEA (3)	80
**7**^d^	**T3P (1.2)**	**1.3**	**EtOAc**	**0.5**	**DIPEA (3)**	**96**
**8**^d^	**T3P (1.2)**	**1.2**	**EtOAc**	**0.5**	**DIPEA (3)**	**93**
9^d^	T3P (1.2)	1.2	EtOAc	0.3	DIPEA (3)	69^e^

a Reactions were carried out on a 0.25 mmol scale unless otherwise specified.

b Yield based on crude mass of **3** (see the SI).

c Run on a 0.5 mmol scale.

d Run on a 1 mmol scale.

e Product precipitated out with heptane. R = activating group; ECF = ethyl chloroformate; 2-EHCF = 2-ethylhexylchloroformate; NMM = *N*-methylmorpholine; DIPEA = Hunig’s base, diisopropylethylamine.

T3P, as an item of commerce, is offered as a 50 w/w % solution in ethyl acetate,^18^ which is a preferred green solvent.^19^ It has also been used extensively on a multikilogram scale^20^ and leads to nontoxic water-soluble byproducts that are low in carbon count following its downstream processing. Intermediates formed using this coupling reagent present no regioselectivity issues, and along with its easy handling during aqueous workup qualifies this reagent from both a practical and sustainable perspective.^13^

Thus, following treatment of **1** with T3P (1.2 equiv) for 1 h, bicyclic proline was added (as its Na salt, **2**; 1.2 equiv) at the same subzero temperatures, along with dilution by addition of EtOAc. The reaction mixture was then allowed to slowly warm to rt with stirring for a period of 20 h.^21^ An *in flask* acidic aqueous workup afforded crude carboxylic acid **3** in 80% isolated yield (see the SI, Table S2 for more information on yield) as a single diastereomer (Table 1, entry 6). Additionally, we found that diluting the reaction mixture to a global concentration of 0.5 M for both steps (i.e., activation of the carboxylic acid and subsequent amide bond formation) led to better stirring of the reaction mixture, which translated into better yields (Table 1, entries 7 and 8).^22^

Subsequent T3P-activated coupling using this material, **3**, was also tested individually in making the second peptide bond. Perhaps not surprisingly given the greater reactivity of primary amine **4**, after combining all reagents in the same pot at subzero temperatures with vigorous stirring, TLC analysis showed complete bond formation after three hours at rt, with full conversion being assessed via TLC and crude ^1^H NMR. A straightforward acidic aqueous work up gave crude nirmatrelvir, which was purified as its MTBE solvate following the Pfizer protocol.^4^

Once the individual steps had been assessed using the same T3P-mediated peptide bond formation, a 3-step, 1-pot sequence was investigated and anticipated to arrive at nirmatrelvir as its MTBE solvate **6**. Carboxylic acid **1** was activated by T3P in the presence of DIPEA at −10 °C over a 1 h period. Bicyclic proline Na salt **2** was then added portion-wise at 0 °C, followed by dilution with anhydrous EtOAc.^23^ The resulting mixture was then warmed slowly to rt with continuous stirring for 20 h.^21^ The mixture was then diluted with a minimal amount of EtOAc, at which point aqueous acid was added to the reaction vessel to remove side-products generated from T3P.

Removal of the aqueous medium from the reaction flask followed by concentration under vacuum (to remove EtOAc) afforded carboxylic acid **3**. Azeotropic drying of **3** with anhydrous toluene removed traces of residual water, followed by introduction of aminonitrile hydrochloride **4** and DIPEA. This mixture was cooled to −10 °C followed by dropwise addition of a 50 w/w % T3P solution in EtOAc. After stirring for five additional minutes, it was then slowly warmed to ambient temperature at which time stirring was continued for an additional three hours. The reaction was then diluted with EtOAc and subjected to an aqueous wash using 1 M HCl. Solvent removal in vacuo afforded crude **5** which was then exposed to 1:10 EtOAc/MTBE. The resultant slurry was washed once with MTBE. Subsequent solvent removal provided nirmatrelvir MTBE solvate **6** in 64% overall yield as a single diastereomer. Furthermore, as disclosed by Pfizer,^24^ the MTBE solvate can eventually be efficiently (95%) carried on to generate the non-solvate form of the API.

**Scheme 2:**
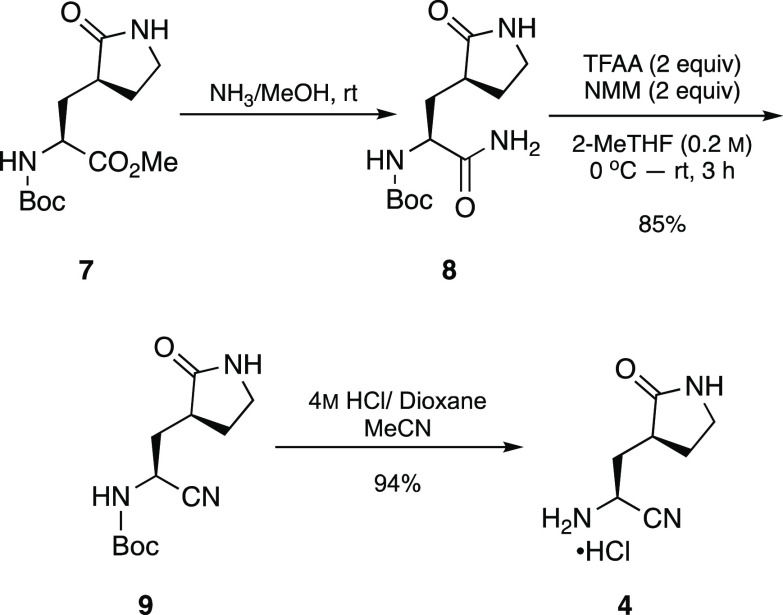
Synthesis of Aminonitrile Hydrochloride salt (**4**)

Aminonitrile hydrochloride salt **4** used in the second peptide coupling (Scheme 2) was prepared from commercially available methyl ester **7** via aminolysis to the known primary amide **8**.^4^ Dehydration of the primary amide to afford the corresponding nitrile was accomplished by treatment with trifluoroacetic anhydride (TFAA) and *N*-methylmorpholine (NMM) in 2-MeTHF, as reported by Shanahan et al.^7^ The *N*-Boc-protected aminonitrile was then subjected to *N*-Boc deprotection using 4 M HCl/dioxane in anhydrous acetonitrile at 0–10 °C, over two hours, as described in our previous route to nirmatrelvir.^9^ The bicyclic proline sodium salt **2** used in the first peptide coupling was prepared following the reported protocol by Pfizer.^4^

Table 2 provides a comparison between this work and Pfizer’s commercial route for multiple key steps. It features a significant improvement in environmental impact, as exemplified by the decrease in the number of pots.^25^ It is also worthy of note that while the overall yields are comparable, this route is limited by the challenges typically associated with small scale sequences, while the comparison data being used has been obtained from reactions run typically on a very large scale.

**Table 2:** Direct Comparison of Routes to the MTBE Solvate **6**

reaction parameter	Pfizer	this work
amide bond formations	uses MsCI, EDCI	***uses T3P***
solvent usage	*i*-PrOAc, MEK, heptane	***EtOAc***
number of pots	4	*1*
overall yield	**65%**^a^	***64%***

a The overall yield is calculated from step 1 through step 4 (MTBE solvate formation) (ref (24)).

## Summary

A straightforward procedure has been developed for the preparation of the targeted drug nirmatrelvir. The 1-pot sequence relies on readily available T3P to effect activation of both carboxylic acid intermediates leading to an overall efficiency of 64%. This route offers a reduced overall carbon footprint associated with the reagents involved, minimal use of organic solvents with opportunities for recycling, and minimal energy usage.

## Data Availability

The data underlying this study are available in the published article and its online Supporting Information.

## Supporting Information Available

Experimental procedures, optimization details, and analytical data (NMR, HPLC, and MS) (PDF)
