# Supplementary material for: A 1-Pot Synthesis of the SARS-CoV-2 Mpro Inhibitor Nirmatrelvir, the Key Ingredient in Paxlovid
Source: Org Lett. 2022 Dec 7;24(49):9049–53. doi: 10.1021/acs.orglett.2c03683 (PMC9764352; doi:10.1021/acs.orglett.2c03683)

Supporting Information

A 1-pot synthesis of the SARS-CoV-2 M^{pro} inhibitor nirmatrelvir, the key ingredient in Paxlovid

Juan C. Caravez, Karthik S. Iyer, Rahul D. Kavthe, Joseph R. A. Kincaid
and Bruce H. Lipshutz*

Department of Chemistry and Biochemistry, University of California, Santa Barbara, CA
93106 USA

Phone : 805-893-2521
Fax : 805-893-8265
Email: lipshutz@chem.ucsb.edu
Website: https://lipshutz.chem.ucsb.edu/

Table of Contents

1. General information S2
2. Synthetic schemes S3
3. Synthetic procedures S4
3.1. Synthesis of starting materials S4
3.1.1. Synthesis of N-trifluoroacetyl t-leucine 1. ..... S4
3.1.2. Synthesis of bicyclic proline, sodium salt 2. ..... S4
3.1.3. Synthesis of aminonitrile hydrochloride 4. ..... S6
3.2. Amide bond coupling en route to carboxylic acid 3 ..... S8
3.3. Amide bond coupling en route to Nirmatrelvir 5. ..... S13
3.4. 3-step, 1-pot synthesis of Nirmatrelvir MTBE solvate 6. ..... S14
4. Determination of Diastereomers by crude ^{1}H NMR S17
5. PMI calculations S18
6. References S20
7. Experimental Data S21

8. ^{1}H, ^{13}C, ^{19}F NMR Spectra of synthesized products..S22

1. General information

Reagents:

Reagents were purchased from Sigma-Aldrich, Combi-Blocks, TCI America Inc., Ambeed Inc., Abovchem, Acros Organics, BLD Pharma, Fischer Scientific, and ChemScene and used without further purification unless noted otherwise.

Chromatography:

Silica gel TLC plates (UV 254 indicator, thickness 200 mm standard grade, glass backed and 230-400 mesh from Merck) were used. The developed TLC plate was analyzed by a UV lamp (254 nm). The plates were further analyzed with the use of an aqueous ceric ammonium molybdate stain or ethanolic vanillin and developed with a heat gun. Flash chromatography was performed using Silicycle Silicaflash® P60 unbonded grade silica.

NMR:

^{1}H, ^{13}C, and ^{19}F NMR were recorded at 25 ºC on either an Agilent Technologies 400 MHz, a Bruker Avance III HD 400 MHz, a Bruker Avance III HD 500 MHz or a Varian Unity Inova 600 MHz spectrometer in D_2O, DMSO-d_6, CD_3OD, or CD_3CN with residual HOD (^{1}H = 4.79 ppm), DMSO (^{1}H = 2.54 ppm, ^{13}C = 40.45 ppm), CH_3OH (^{1}H = 3.31 ppm, ^{13}C = 49.15 ppm), and CH_3CN (^{1}H = 1.94 ppm, ^{13}C = 1.39 ppm) as the internal standard. Chemical shifts are reported in parts per million (ppm). The data presented will be reported as follows; chemical shift, multiplicity (s = singlet, bs = broad singlet, d = doublet, dd = doublet of doublet, t = triplet, q = quartet, quin = quintet, m = multiplet), coupling constant (if applicable), and integration.

HPLC:

HPLC-grade solvents were obtained from Fischer Scientific.

HPLC analysis was performed on an Agilent 1260 series HPLC with a Lux 5u Cellulose-2 column (250 x 4.6 mm, 5 μm) at a flow-rate of 1.25 mL/min using 50% v/v isopropanol/hexanes. Spectral data at 210 nm were collected.

2. Synthetic schemes

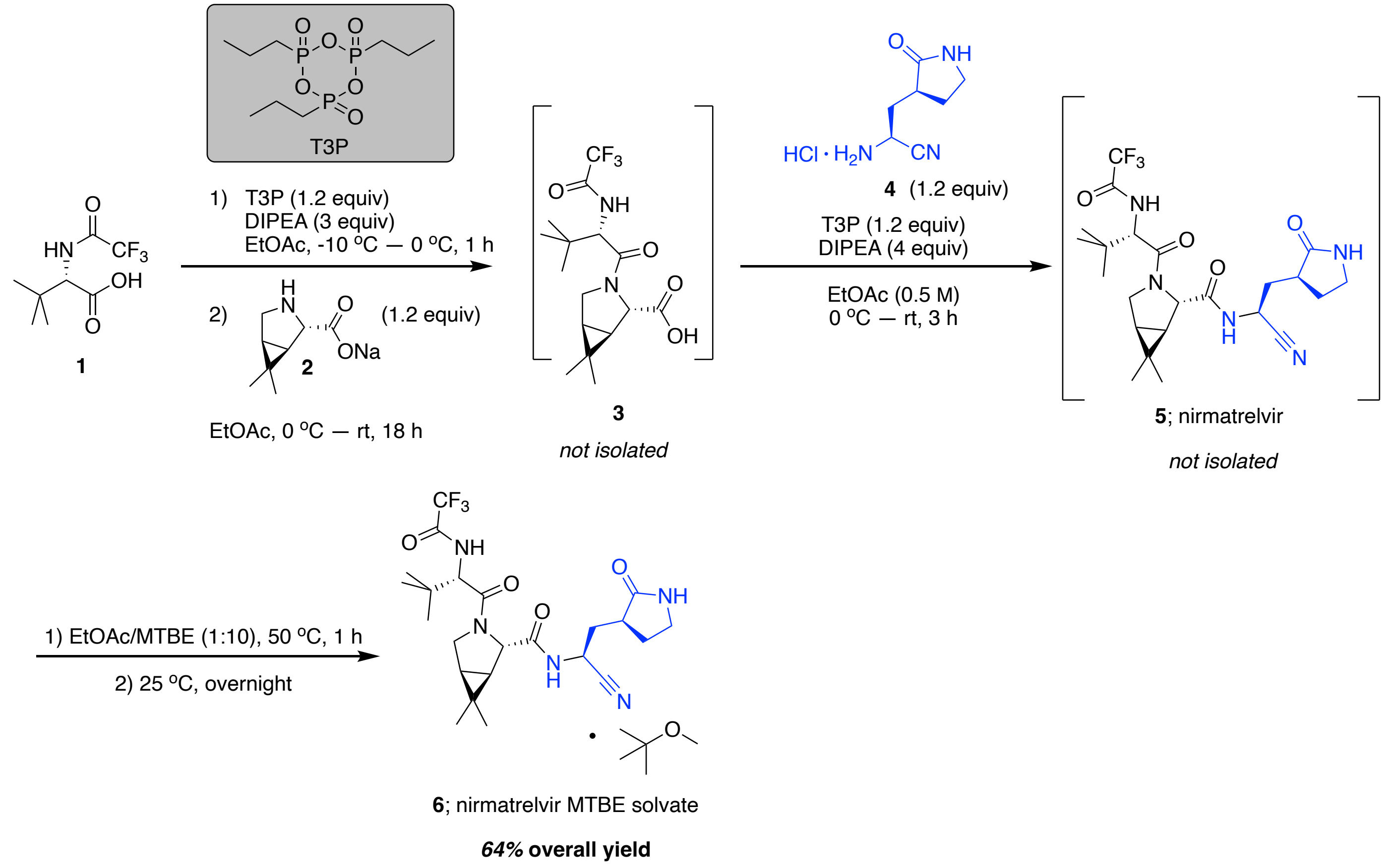

Scheme S1: Overall synthesis of nirmatrelvir MTBE solvate (**6**).

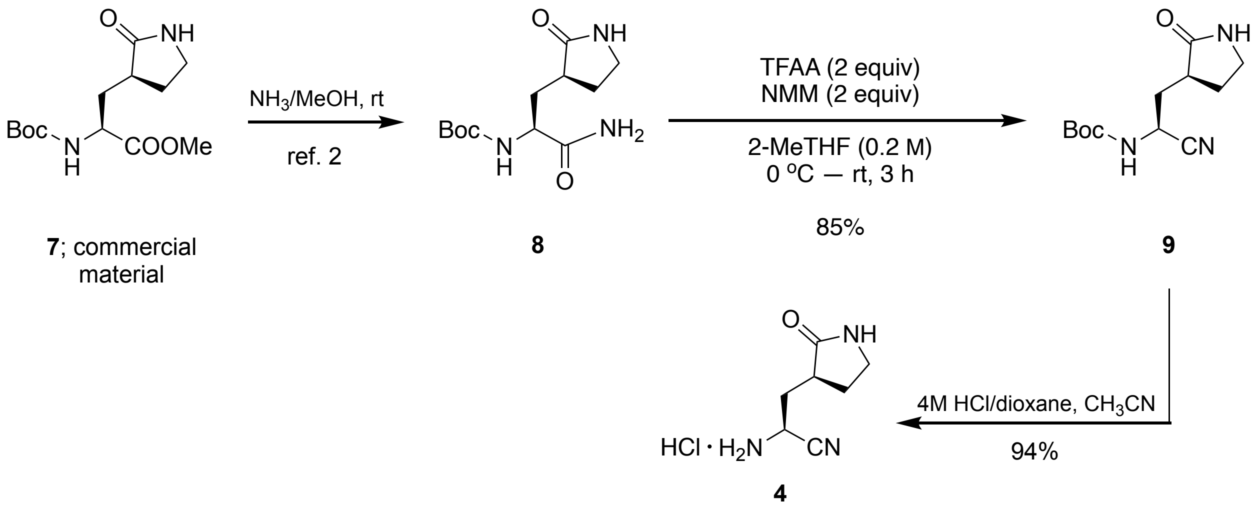

Scheme S2: Synthesis of aminonitrile hydrochloride (**4**).

3. Synthetic procedures

3.1. Synthesis of starting materials

3.1.1. Synthesis of *N*-trifluoroacetyl *t*-leucine **1**.

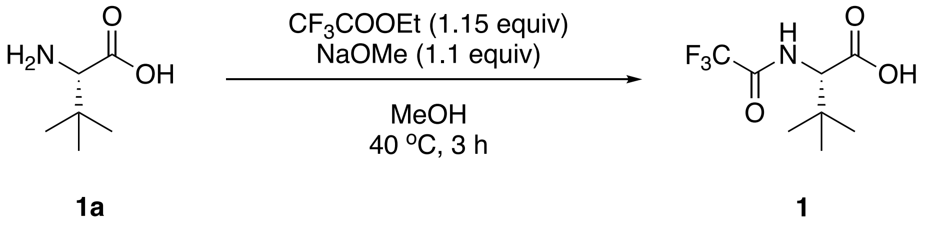

Scheme S3: Synthesis of *N*-TFA *t*-leucine (**1).**

This procedure was recently disclosed by Pfizer.[1]

An oven-dried 100 mL round bottom flask with a PTFE-coated magnetic stir-bar was charged with L-*t*-leucine (20 mmol, 2.62 g) (**1a**) in anhydrous methanol (5.3 mL) (stored over 3Å molecular sieves) followed by the addition of a 25 wt % solution of sodium methoxide in methanol (22 mmol, 6 mL). Ethyl trifluoroacetate (1.15 equiv, 2.7 mL) was added dropwise and the reaction mixture was stirred at 40 °C in an oil bath for 3 h. Upon completion, the reaction mixture was allowed to cool to rt, concentrated *in vacuo* to remove most of the methanol, then acidified to pH 2 using a 1 M aqueous solution of HCl. The aqueous phase was extracted twice with EtOAc, and the combined organic layers were washed with brine, dried over anhydrous Na_2SO_4, and then concentrated *in vacuo* to afford *N*-trifluoroacetyl *t*-leucine **1** (4 g, 88% yield) as a white solid. This material was dried azeotropically with toluene at 40 °C and used without further purification.

3.1.2. Synthesis of bicyclic proline, sodium salt **2**.

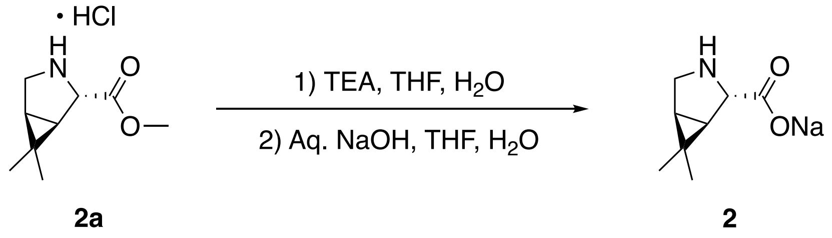

Scheme S4: Synthesis of sodium (1*R*,2*S*,5*S*)-6,6-dimethyl-3-azabicyclo[3.1.0]hexane-2-carboxylate (**2**).

This procedure was recently disclosed by Pfizer.[1]

To a 6-dram vial with a PTFE coated magnetic stir-bar was added (1*R*,2*S*,5*S*)-6,6-dimethyl-3-azabicyclo[3.1.0]hexane-2-carboxylate hydrochloride **2a** (12.2 mmol, 2.5 g) followed by the addition of tetrahydrofuran (10 mL), water (2.5 mL) and triethylamine (1.5 equiv, 18.3 mmol, 3.8 mL). The mixture was stirred at 25 °C for 1 h, then stirring was halted and the layers were separated. In a separate 100 mL round-bottom flask, 28 w/w % of aqueous sodium hydroxide (1.03 equiv, 12.56 mmol, 1.74 mL) and THF (27 mL) were added with stirring at 40 °C. Ca. 25% of the solution of free-based **2a** in THF from the layer separation was added to the mixture and this solution was seeded with sodium (1*R*,2*S*,5*S*)-6,6-dimethyl-3-azabicyclo[3.1.0]hexane-2-carboxylate (**2**) (previously prepared using the same protocol). This mixture was held at 40 °C for 30 min and the remaining ca. 75% of the organic layer was added dropwise while stirring at 40 °C. The mixture was stirred for 16 h at 40 °C, and then cooled slowly to rt and held at rt for 3 h. The resulting solid was isolated by filtration and subsequently washed by a solution of 5 v/v % water in THF. The solid material was then azeotropically dried with toluene (3 x 15 mL) at 40 °C to afford sodium (1*R*,2*S*,5*S*)-6,6-dimethyl-3-azabicyclo[3.1.0]hexane-2-carboxylate (**2**) (2.1 g, 97% yield) as a white amorphous solid. The purity of this material was determined by quantitative ^{1}H NMR (20 s relaxation delay) in CD_3OD with trimethoxybenzene as the internal standard and was found to be 85%.

Note: Depending on the purity of the commercially available starting material ***2a****, the purity of the corresponding sodium salt* ***2*** *can vary.*

Alternatively, we tried forming the free base of ***2a*** *by using a 1:1 mixture of DCM and 10% aq.* Na_2CO_3 *and stirring it at rt for 30 min. The organic layer was concentrated in vacuo and the purity was checked by quantitative* ^{1}*H NMR analysis. It was found to be 93% pure, showing that most of the impurities are water soluble. However, from a green aspect, we use THF and* Et_3N *for freebasing* ***2a****, which might result in the impurities being carried forward in the subsequent step, due to miscibility of THF with water.*

3.1.3. Synthesis of aminonitrile hydrochloride **4.**

Primary amide starting material **8** was prepared as previously reported.[2]

- Procedure for amide dehydration of primary amide **8** to afford nitrile **9**:

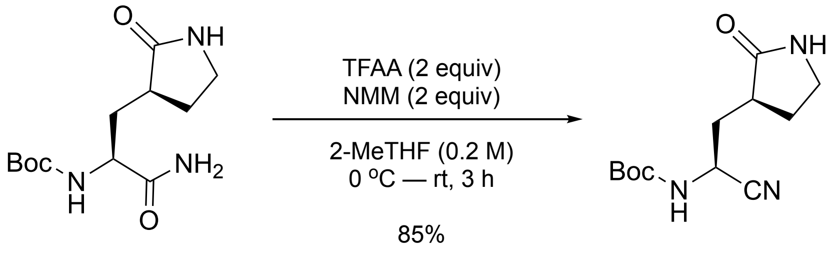

8 **9**

Scheme S5: Synthesis of *N*-Boc-protected nitrile (**9**).

This procedure was reported recently by Shanahan *et. al*.[3]

To a stirred solution of primary amide **8** (1 mmol, 271.3 mg) in anhydrous 2-MeTHF (0.2 M, 5 mL) (used as is from a *sure-seal* bottle from Sigma-Aldrich) at 0 °C, was added *N*-methylmorpholine (2 equiv, 220 μL) followed by dropwise addition of trifluoroacetic anhydride (2 equiv, 278 μL) at 0 °C. The reaction mixture was stirred at 0 °C for 15 min and then at rt for 3 h. Upon completion, the reaction was quenched by addition of brine (10 mL) and extracted twice with EtOAc. The combined organic layers were washed with a mixture of 1 M HCl (5 mL) followed by a mixture of saturated sodium bicarbonate (5 mL). Finally, the organic layer was washed with brine (5 mL), dried over anhydrous sodium sulfate, and concentrated under reduced pressure to obtain **9** (85% yield, 215 mg) as a white solid.

Note: Depending on the source of the commercial material 7, the primary amide ***8*** *and the amino nitrile* ***9*** *can have a slight off-white color due to certain impurities that do not affect the reaction outcome. These impurities can be removed by suspending crude* ***9*** *in EtOAc (due to very low solubility of* ***9*** *in this solvent) and removing the supernatant by centrifugation, to afford pure* ***9*** *as a white solid.*

- *N*-Boc Deprotection *en route* to amine hydrochloride salt **4**.[4]

N-Boc Deprotection could be effected using HCl in organic solvents; however, the *N*-Boc deprotected nitrile **9** was susceptible to hydrolysis due to the presence of adventitious moisture, leading to a mixture of products (nitrile **4**, carboxylic acid, and primary amide; as disclosed in our previous report).[4]

- Protocol for the *N*-Boc deprotection to afford amine hydrochloride salt **4**:

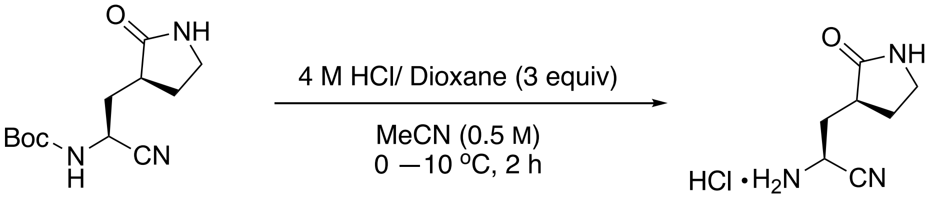

9 **4**

Scheme S6: Synthesis of aminonitrile HCl salt (**4**).

N-Boc Deprotection to afford aminonitrile hydrochloride salt **4** was performed according to a prior literature report.[4] Starting material **9** was azeotropically dried by suspending it in anhydrous toluene (obtained from a solvent purification system) and removing solvent *in vacuo* at <60 °C a total of three times. To an oven-dried 6-dram vial equipped with a PTFE-coated magnetic stir bar was added *N*-Boc protected nitrile **9** (1 equiv, 1.777 mmol, 450 mg) and anhydrous CH_3CN (0.5 M, 3.55 mL) (stored over 3Å molecular sieves) generating a cloudy suspension, after which the mixture was chilled in an ice bath. To the vial was added 4 M HCl/dioxane dropwise via syringe (3 equiv, 5.33 mmol, 1.33 mL) whereupon starting material immediately went into solution. The reaction was allowed to warm to rt and stir for 2 h. As the reaction progressed, product precipitated as a white solid, and was collected via filtration and washed with ice-cold Et_2O to afford **4** (322.1 mg, 96% yield) as a white solid. If removal of hydrolysis by-products is required, the solid can be dissolved in minimal anhydrous MeOH (obtained by refluxing with Mg turnings and I_2 and distilling over 3Å molecular sieves) with heating. The solution is then cooled to 0-5 °C in an ice

bath, after which ice-cold Et_2O was added dropwise until material stopped precipitating. The precipitate was collected via filtration and dried under vacuum to afford **4**.

3.2. Amide bond coupling *en route* to carboxylic acid **3**

Optimization of the first amide bond coupling began by screening different methods for activating carboxylic acid **1**. Initially, activation via formation of the acyl chloride using thionyl chloride ($SOCl_2$) was screened with the use of lithium (1*R*,2*S*,5*S*)-6,6-dimethyl-3-azabicyclo[3.1.0]hexane-2-carboxylate (**2b**) (prepared according to a protocol previously reported by Pfizer)[1] as the amine coupling partner. Solvents (including aqueous surfactant solutions using TPGS-750-M),[6] bases, and temperatures were screened.

- Procedure for optimization using thionyl chloride.

  To an oven-dried 1-dram vial with a PTFE-coated magnetic stir-bar was added carboxylic acid **1** (0.25 mmol, 57 mg), followed by the addition of solvent (0.5 M). The vial was purged with argon and cooled to -10 °C using an acetone-ice bath and thionyl chloride was added dropwise with stirring. The reaction mixture was allowed to stir for 3-4 h at -10 °C. Upon completion, excess thionyl chloride was removed under high vacuum and the mixture was diluted with solvent. In a separate flame dried 1-dram vial with a magnetic stir-bar was added the lithium salt **2b** (1 equiv, 39 mg). The acid chloride mixture was added dropwise to the vial containing **2b** at -10 °C and the mixture was allowed to warm up to rt and stir for 20 h. Upon completion, the reaction was further diluted with solvent and was sequentially washed with 10% aqueous citric acid solution (1.5 mL) and water (1.5 mL) . The organic layer was concentrated to afford crude carboxylic acid **3**.

Table S1: Initial optimization of amide bond formation using the lithium salt of the amino acid.

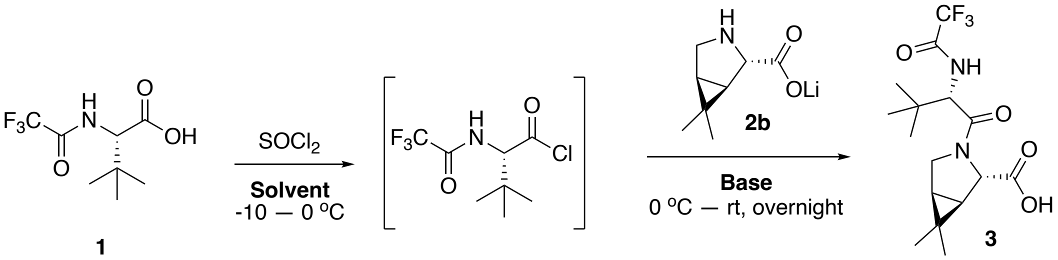

Entry[a]	$SOCl_2$ equiv.	Solvent	Concentration (M)	Base (equiv)	Yield[b]
1	5	DCM	0.5	DIPEA (1.5 eq)	traces
2	15	THF	0.5	DIPEA (1.5 eq)	15%
3	3	toluene	0.5	DIPEA (2 eq)	40%
4	1.25	*i*-PrOAc	1.5	DIPEA (1.2 eq)	20%
5	2	-	neat	DIPEA (1.2 eq)	traces

[a] Reactions were carried out on a 0.25 mmol scale; [b] yield based on mass of crude **3**.

- Optimization of the amide bond using other coupling reagents

Due to the extremely sensitive nature of acid chlorides to moisture and their difficulty in handling, other coupling reagents were screened for activation of the carboxylic acid **1** (see Table S2). Moreover, the lithium salt **2b** was replaced by the sodium salt **2** due to the better stirring properties of the latter.

- Procedure for optimization of the amide bond using other coupling reagents.

To an oven-dried 1-dram vial with a PTFE-coated magnetic stir-bar was added carboxylic acid **1** (0.25 mmol, 57 mg, 1 equiv), followed by the addition of solvent (0.5 M, 0.5 mL). The vial was purged with argon and cooled to -10 °C using an acetone-ice bath, then coupling reagent was added dropwise with stirring (TsCl was added in one portion). The reaction mixture was allowed to stir for 1 h at -10 °C. Upon completion, the mixture was diluted with solvent. In a separate flame dried 1-dram vial with a magnetic stir-bar was

added the sodium salt **2.** The mixed anhydride solution was added dropwise to the vial containing **2** at -10 °C and the mixture was allowed to warm up to rt and stir for 20 h. Upon completion, the reaction was further diluted with solvent and was washed with 1 M HCl solution and then with water. The organic layer was concentrated under reduced pressure to afford crude carboxylic acid **3**. (*In case of THF as solvent, the reaction mixture was concentrated and re-diluted with EtOAc prior to work-up).*

Table S2: Optimization of amide bond formation using different coupling reagents.

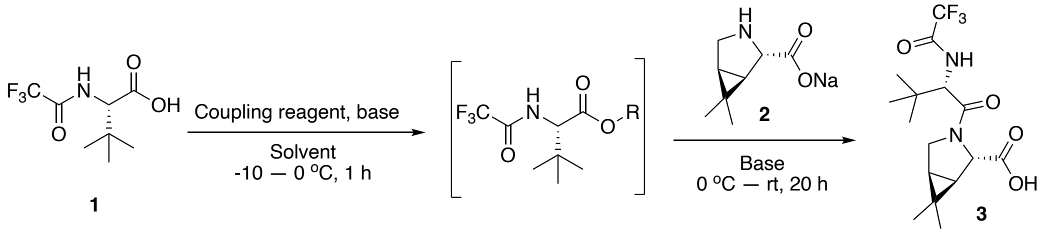

Entry[a]	Coupling reagent (equiv)	Amine 2 (equiv)	Solvent	Concentration (M)	Base (equiv)	Yield[b]
1	ECF[c] (1)	0.83	EtOAc	0.5	NMM (1)	40%
2	ECF (1.2)	1.2	THF	0.5	NMM (1)	45%
3	ECF (1.2)	1.2	*i*-PrOAc	0.5	NMM (1.2)	27%
4	ECF (1.2)	1.2	2-MeTHF	0.5	NMM (1.2)	60%
5	ECF (1.2)	1.2	2-MeTHF	0.5	Et_3N (1.2)	messy
6	TsCl (1.2)	1.2	THF	0.4	DIPEA (3), DMAP (cat.)	71%
7	MsCl (1.2)	1.2	*i*-PrOAc	0.5	Et_3N (2)	57%
8	CDI (1.2)	1.2	THF	0.5	-	59%
9	PivCl (1.2)	1.2	THF	0.3	Et_3N (1.2)	53%
10	PivCl (1.2)	1	*i*-PrOAc/2 wt % TPGS-750-M/H_2O	0.5-0.2	Et_3N (3) /$NaHCO_3$ (3)	messy
11	T3P (1.2)	1.2	EtOAc	0.3	DIPEA (2)	73%
12[d]	T3P (1.2)	1.2	EtOAc	1.4-0.8	DIPEA (3)	80%
13[d]	T3P (1.2)	1.2	EtOAc	1.4-0.8	DIPEA (3)	79%[e]
14[d, f]	T3P (1.2)	1.2	EtOAc	0.5	DIPEA (3)	messy
**14[d]**	**T3P (1.2)**	**1.3**	**EtOAc**	**0.5**	**DIPEA (3)**	**96%**
**15[d]**	**T3P (1.2)**	**1.5**	**EtOAc**	**0.5**	**DIPEA (3)**	**96%**
**16[d]**	**T3P (1.2)**	**1.2**	**EtOAc**	**0.5**	**DIPEA (3)**	**93%**
17[g]	T3P (1.2)	1.2	EtOAc	0.5	DIPEA (3)	93%
18[g]	T3P (1.2)	1.2	EtOAc	0.3	DIPEA (3)	69%[h]

[a] Reactions carried out on a 0.25 mmol scale unless otherwise noted; [b] yield based on mass of crude **3**; [c] ECF= ethyl chloroformate; [d] Reaction carried out on a 0.5 mmol scale; [e] Product isolated by flash chromatography; [f] 10 mol % DMAP was used as an additive; [g] Reaction carried out on a 1 mmol scale; [h] precipitated with heptane.

*Note: These reactions were carried out to confirm the quality of amide bond formation. The crude product has been telescoped into the next step to make the final MTBE solvate **6** (vide infra).*

- Optimized protocol for amide bond coupling *en route* to carboxylic acid **3**:

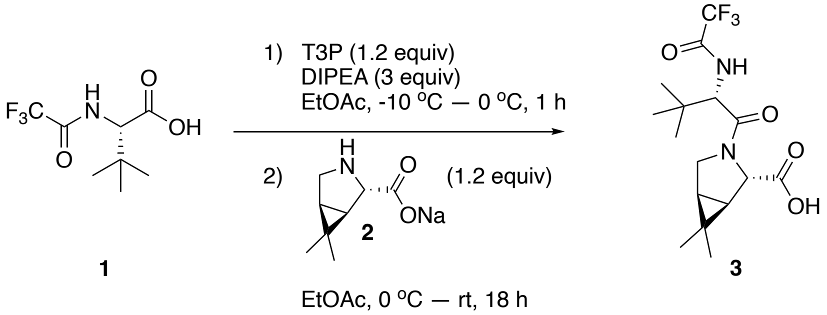

Scheme S7: Amide coupling to afford carboxylic acid (**3**).

To an oven-dried 6-dram vial with a PTFE coated magnetic stir-bar was added (*S*)-3,3-dimethyl-2-(2,2,2-trifluoroacetamido)butanoic acid (**1;** 1 mmol, 227 mg) followed by addition of anhydrous EtOAc (0.5 M, 1.6 mL) (stored over 4Å molecular sieves) and DIPEA (3 equiv, 3 mmol, 522 µL) and the solution was cooled to -10 °C using an acetone-ice bath. The mixture was stirred for 5 min at this temperature at which time a 50 w/w % solution of propane phosphonic acid anhydride (T3P) in EtOAc (1.2 equiv, 1.2 mmol, 716 µL) was added dropwise with stirring and the mixture was stirred for 1 h while maintaining the temperature at -10 °C. Upon completion of mixed anhydride formation, the bicyclic proline Na salt **2** (1.2 equiv, 1.2 mmol, 212.6 mg) was added portion-wise at a temperature of 0 °C. The mixture was allowed to warm up eventually to rt and stirred for 18 h. Upon completion, the reaction was diluted with EtOAc (2 mL) and washed with a mixture of 1 M aqueous HCl solution (2 mL), and brine (1 mL). The aqueous layer was extracted 3 times with EtOAc (2 mL), and the combined organic layers were concentrated to afford crude carboxylic acid **3** as an off white solid (93% yield, 338.5 mg). This material was used without purification in the subsequent step. The purity of this material was determined by quantitative ^{1}H NMR (20 s relaxation delay) in DMSO-d_6 using trimethoxybenzene as an internal standard, and was found to be 78% pure.

3.3. Amide bond coupling *en route* to nirmatrelvir **5**.

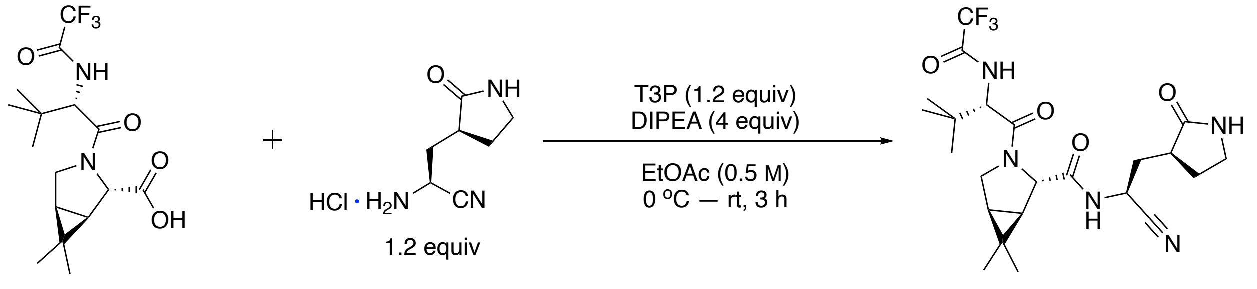

Scheme S8: Amide coupling to afford nirmatrelvir (**5**).

- Procedure for amide bond coupling *en-route* to Nirmatrelvir **5**.

To an oven-dried 6-dram vial with a PTFE-coated magnetic stir-bar, pure carboxylic acid **3** (1 mmol, 364.4 mg), previously synthesized from Pfizer's protocol[1], was added followed by the addition of the aminonitrile hydrochloride **4** (1.2 equiv, 1.2 mmol, 228 mg), DIPEA (4 equiv, 4 mmol, 700 µL) and anhydrous EtOAc (0.5 M, 1.6 mL), and the solution was cooled to -10 °C using an acetone-ice bath. The mixture was stirred for 5 min at this temperature at which time a 50 w/w % solution of propane phosphonic acid anhydride (T3P) in EtOAc (1.2 equiv, 1.2 mmol, 716 µL) was added dropwise under stirring and the resulting reaction mixture was stirred for 30 min, then allowed to warm up to rt and stirred for 3 h. On completion of the reaction (as monitored by TLC), the mixture was diluted with EtOAc (2 mL) and washed with a mixture 1 M aqueous HCl solution (2 mL) and brine (1 mL), and the aqueous layer was extracted 3 times with EtOAc (2 mL). The combined organic layers were concentrated to afford crude Nirmatrelvir (**5)** as an off white solid (98% yield, 490 mg, 95% purity by HPLC).

3.4. 3-Step, 1-pot synthesis of nirmatrelvir MTBE solvate **6**.

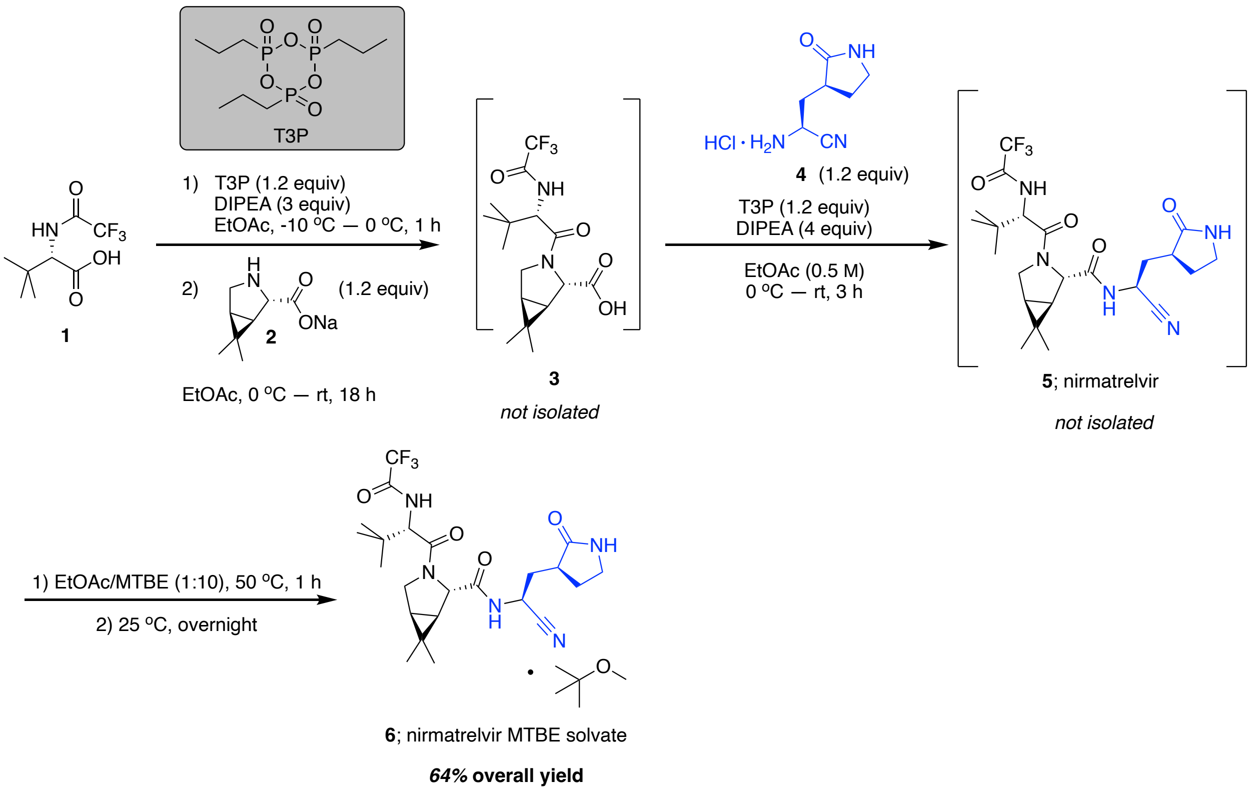

Scheme S9: 1-pot synthesis of Nirmatrelvir MTBE solvate (**6**).

Step 1: Amide bond formation to afford carboxylic acid 3.

To an oven-dried 6-dram vial with a PTFE coated magnetic stir-bar was added (*S*)-3,3-dimethyl-2-(2,2,2-trifluoroacetamido)butanoic acid (**1,** 1 mmol, 227 mg) followed by addition of EtOAc (0.5 M, 1.6 mL) and DIPEA (3 equiv, 3 mmol, 522 µL) and the solution was cooled to -10 °C using an acetone-ice bath. The mixture was stirred for 5 min at this temperature at which time a 50 w/w % solution of propane phosphonic acid anhydride (T3P) in EtOAc (1.2 equiv, 1.2 mmol, 716 µL) was added dropwise with stirring and the mixture was stirred for 1 h while maintaining the temperature at -10 °C. Upon completion of mixed anhydride formation, the bicyclic proline Na salt **2** (1.2 equiv, 1.2 mmol, 212.6 mg) was added portion-wise at a temperature of 0 °C. The mixture was allowed to warm to rt and stirred for 18 h. Upon completion, the reaction was diluted with EtOAc (2 mL) and washed with a mixture of 1 M aqueous HCl solution (2 mL), and brine (1 mL). The aqueous layer was extracted 3 times with EtOAc (2 mL), and the combined organic

layers were concentrated to afford crude carboxylic acid **3** as a white to off white solid. This material was subjected to azeotropic drying with toluene (2 x 6 mL) at 40 ºC to remove any residual water present and was used without purification in the subsequent step.

Step 2: Amide bond coupling to afford nirmatrelvir 5.

The material from the previous step was subjected to aminonitrile hydrochloride **4** (1.2 equiv, 1.2 mmol, 228 mg) and DIPEA (4 equiv, 4 mmol, 700 µL) followed by the addition of anhydrous EtOAc (0.5 M, 1.6 mL), and the solution was cooled to -10 ºC using an acetone-ice bath. The mixture was stirred for 5 min at this temperature whereupon a 50 w/w % solution of propane phosphonic acid anhydride (T3P) in EtOAc (1.2 equiv, 1.2 mmol, 716 µL) was added dropwise under stirring and the resulting reaction mixture was stirred for 30 min at this temperature, at which time it was allowed to warm to rt and stirred for 3 h. On completion of the reaction (as monitored by TLC), the mixture was diluted with EtOAc (2 mL) and washed with a mixture of 1 M aqueous HCl solution (2 mL), and brine (1 mL) and the aqueous layer was extracted 3 times with EtOAc (2 mL). The combined organic layers were concentrated *in vacuo* to afford crude nirmatrelvir (**5**) as an off white solid (97% yield, 485 mg). This was taken directly into the MTBE solvate formation in the same vial.

Step 3: MTBE solvate formation to afford nirmatrelvir MTBE solvate 6.

The crude material from step 2 above was subjected to a 1:10 mixture of EtOAc/MTBE (0.23 mL EtOAc, 2.4 mL MTBE) and the reaction was stirred at 50 ºC for 1 h whereupon it was allowed to cool to rt and stir overnight. The resultant slurry was allowed to stand for 1 h at rt without stirring, whereupon the solvent was carefully removed using a pipette. The solid was washed by addition of *t*-butyl methyl ether (MTBE, 5 mL) and stirred for 10 min. When the solid had settled at the bottom, the solvent was removed via pipette. The resulting solid was dried under high vacuum to afford nirmatrelvir MTBE solvate **6** as a white solid (64% yield over 3 steps, 375 mg, >99.5% purity by HPLC, see figure S3).

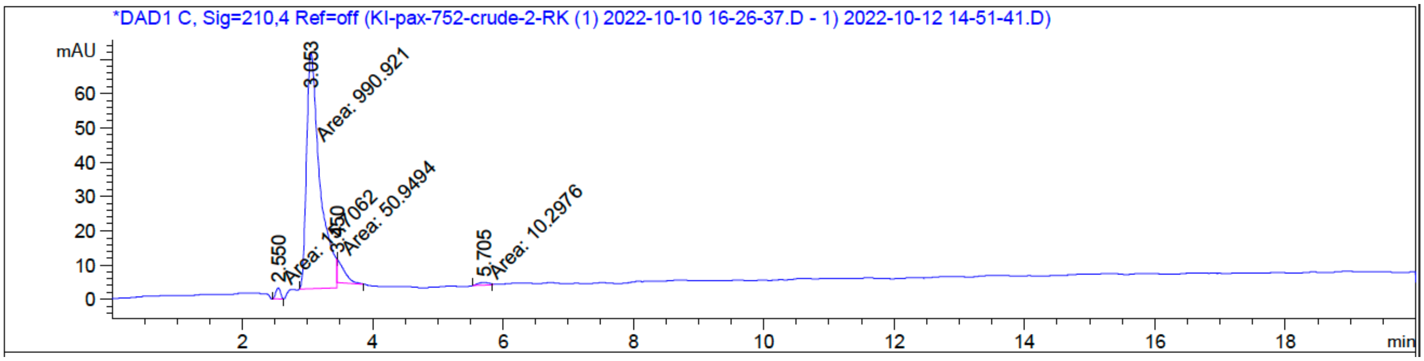

Signal 3: DAD1 C, Sig=210,4 Ref=off
Signal has been modified after loading from rawdata file!

Peak #	RetTime [min]	Type	Width [min]	Area [mAU*s]	Height [mAU]	Area %
1	2.550	MM T	0.0812	15.70617	3.22465	1.4708
2	3.053	MM T	0.2399	990.92090	68.84358	92.7938
3	3.450	MM T	0.1239	50.94941	6.85379	4.7711
4	5.705	MM T	0.2086	10.29764	8.22629e-1	0.9643
Totals :				1067.87411	79.74464	

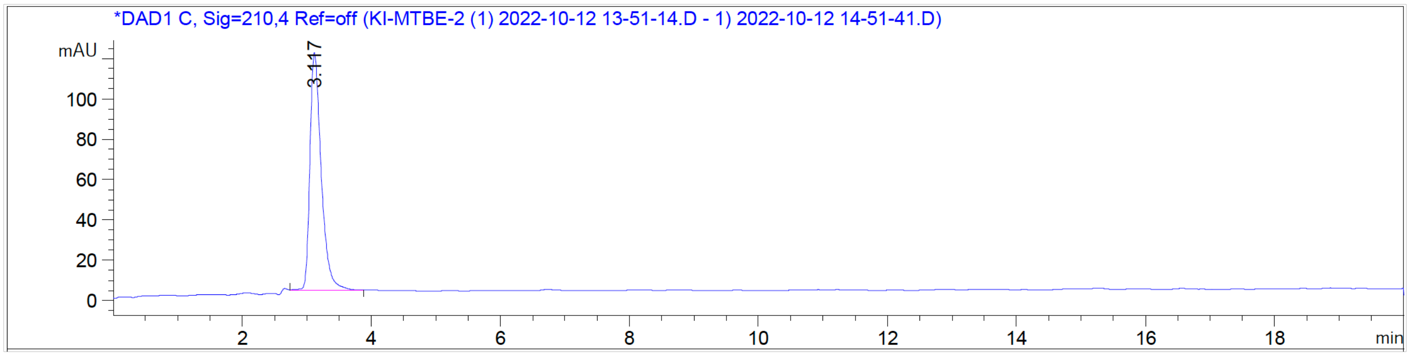

Signal 3: DAD1 C, Sig=210,4 Ref=off
Signal has been modified after loading from rawdata file!

Peak #	RetTime [min]	Type	Width [min]	Area [mAU*s]	Height [mAU]	Area %
1	3.117	BB	0.1816	1430.69189	117.89877	100.0000
Totals :				1430.69189	117.89877	

Figure S3: HPLC analysis of a) crude nirmatrelvir and; b) nirmatrelvir MTBE solvate **6** respectively via chiral HPLC measured at 210 nm (see SI section 1, HPLC method 1).

4. Determination of Diastereomers by crude ^{1}H NMR

The diastereomer of nirmatrelvir was isolated via column chromatography as mentioned in our previous publication[4] and subjected to HRMS (*m/z* calcd for $C_{23}H_{32}F_{3}N_{5}O_{4}+H^{+}$: 500.2479 [*M*+H]+: found 500.2474), which confirmed that the material was a diastereomer of nirmatrelvir **5**. It should be noted that we do not currently know which stereocenter epimerizes, but our best guess would be the center alpha- to the nitrile group.

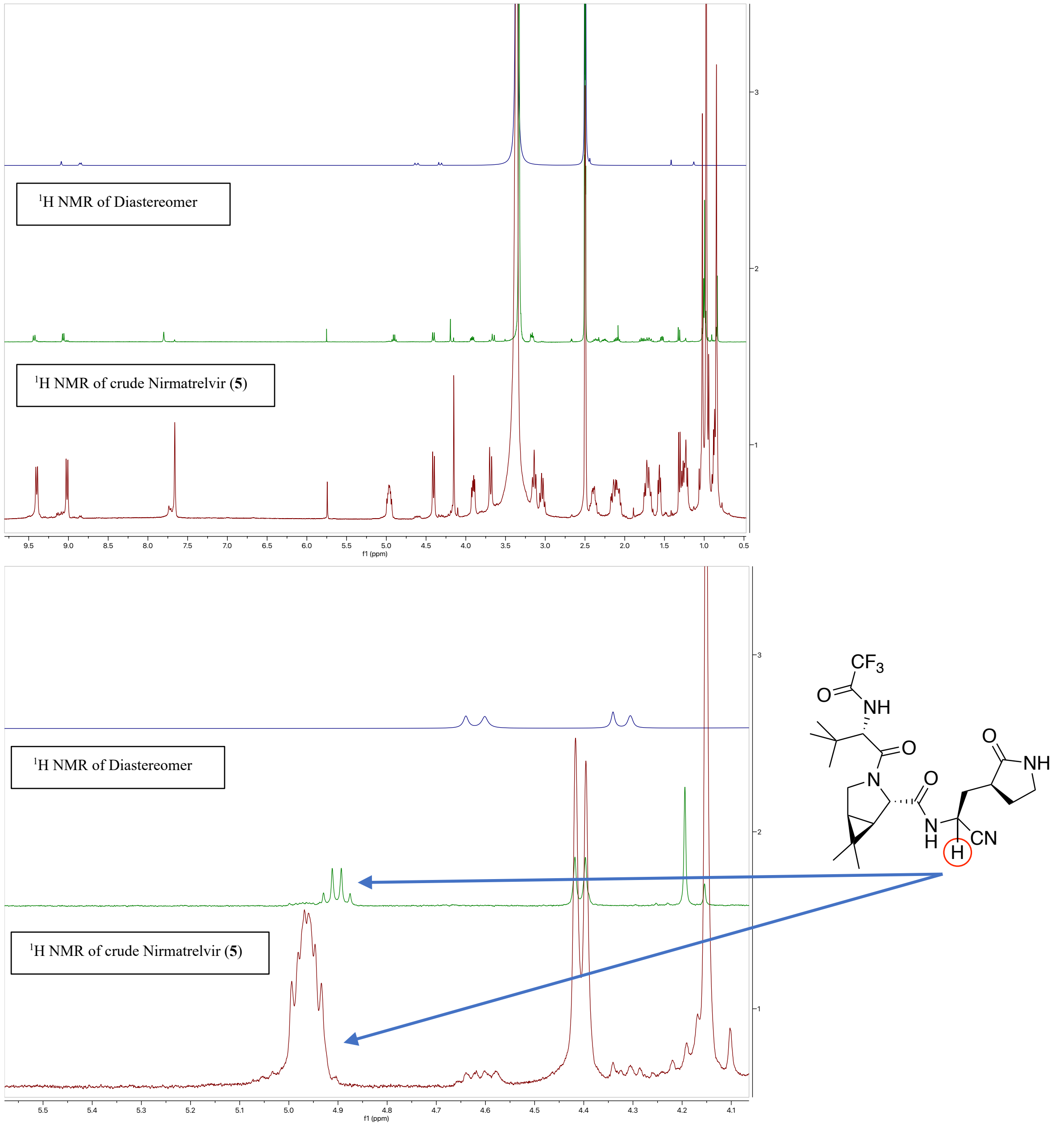

Figure S4: a) Determination of the presence of diastereomers via crude ^{1}H NMR analysis; b) Blown up section of the proton adjacent to the nitrile group.

As exemplified by chiral HPLC and the above-mentioned NMR spectra, the presence of diastereomers in the crude API was not observed.

5. PMI calculations

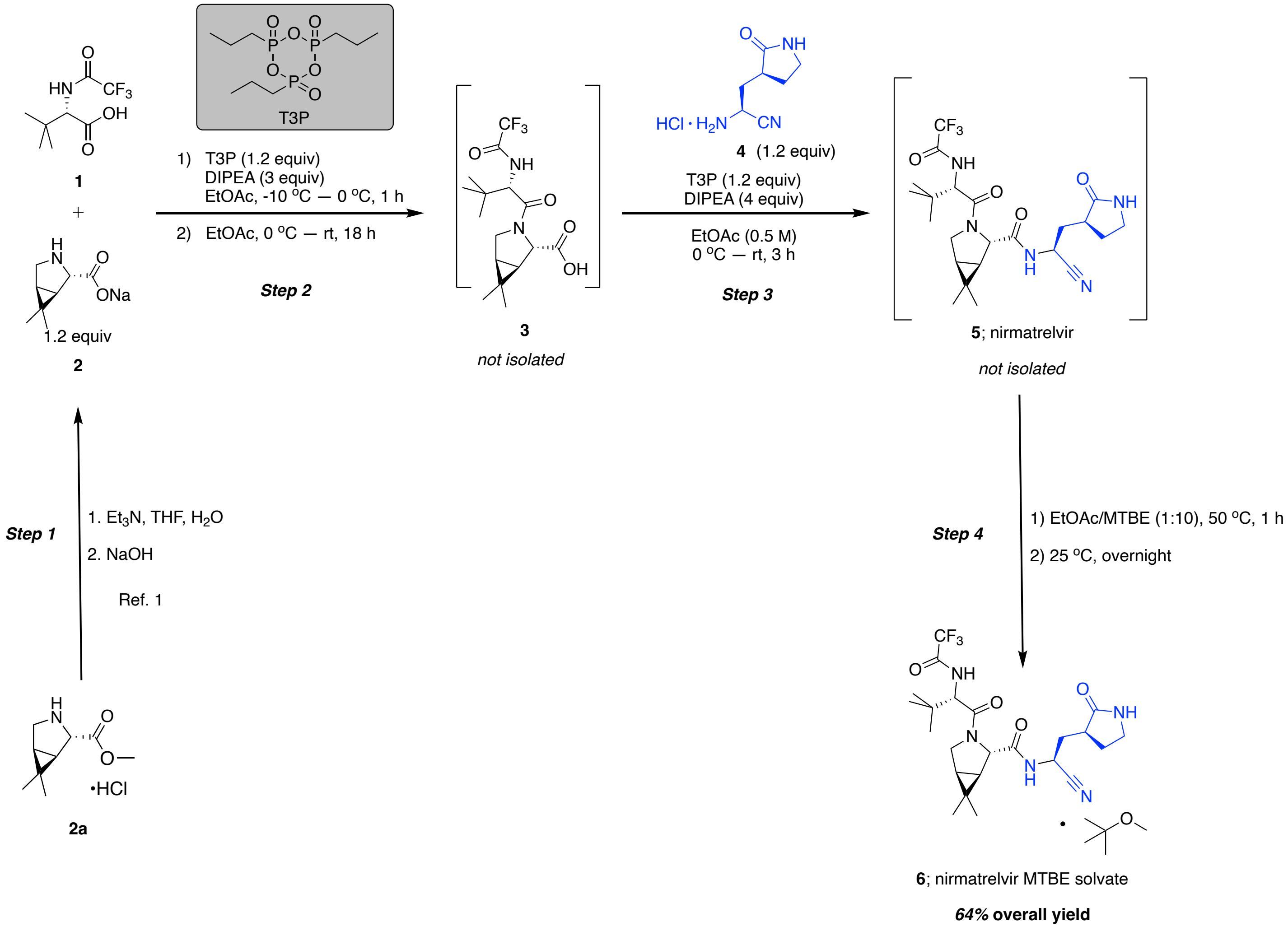

Scheme S10: Overall synthesis of nirmatrelvir MTBE solvate (**6**) used for PMI calculations.

To compare this work's PMI to Pfizer's commercial route, an additional step, i.e., the conversion of **2a** to **2** has also been considered in the following calculations.

Step 1

- **2a**: 0.275 g
- Et_3N: 0.2 g
- THF: 3.64 g

- Water: 0.27 g
- Sodium methoxide: 0.072 g
- Methanol: 0.185 g

Step 2

- *N*-TFA-tert leucine **2**: 0.227g
- T3P: 0.381 g
- EtOAc: 0.381 g
- DIPEA: 0.387 g
- EtOAc: 1.44 g
- Toluene: 10.4 g

Step 3

- **4**: 0.228 g
- T3P: 0.381 g
- EtOAc: 0.381 g
- DIPEA: 0.517 g
- EtOAc: 1.44 g

Step 4

- MTBE: 5.55 g
- EtOAc: 0.2 g

Total amount of MTBE solvate produced: 0.375 g

PMI: Total raw materials / Total amount of API produced

PMI = 26.54 / 0.375 = **70**

6. References

1. Owen, D. R.; Pettersson, M. Y.; Reese, M. R.; Sammons, M. F.; Tuttle, J. B.; Verhoest, P. R.; Wei, L.; Yang, Q.; Yang, X. Nitrile-Containing Antiviral Compounds. U.S. Patent US 20220062232A1, March 3, 2022.
2. Owen, D. R.; Allerton, C. M. N.; Anderson, A. S.; Aschenbrenner, L.; Avery, M.; Berritt, S.; Boras, B.; Cardin, R. D.; Carlo, A.; Coffman, K. J.; Dantonio, A.; Di, L.; Eng, H.; Ferre, R.; Gajiwala, K. S.; Gibson, S. A.; Greasley, S. E.; Hurst, B. L.; Kadar, E. P.; Kalgutkar, A. S.; Lee, J. C.; Lee, J.; Liu, W.; Mason, S. W.; Noell, S.; Novak, J. J.; Obach, R. S.; Ogilvie, K.; Patel, N. C.; Pettersson, M.; Rai, D. K.; Reese, M. R.; Sammons, M. F.; Sathish, J. G.; Singh, R. S. P.; Steppan, C. M.; Stewart, A. E.; Tuttle, J. B.; Updyke, L.; Verhoest, P. R.; Wei, L.; Yang, Q.; Zhu, Y. An Oral SARS-CoV-2 M pro Inhibitor Clinical Candidate for the Treatment of COVID-19. *Science* **2021**, *374*, 1586–1593.
3. Shanahan, C. S.; Kadam, A. L.; Chiranjeevi, B.; Nunes, A. A.; Jayaraman, A.; Ahmad, S.; Aleshire, S. L.; Donsbach, K. O.; Gupton, B. F.; Nuckols, M. C. Efforts to Develop a Cost-Effective and Scalable Synthetic Process for Nirmatrelvir. *ChemRxiv* **2022**. DOI: 10.26434/chemrxiv-2022-cn0k1.This content is a preprint and has not been peer-reviewed.
4. Kincaid, J. R. A.; Caravez, J. C.; Iyer, K. S.; Kavthe, R. D.; Fleck, N.; Aue, D. H.; Lipshutz, B. H. A Sustainable Synthesis of the SARS-CoV-2 Mpro Inhibitor Nirmatrelvir, the Active Ingredient in Paxlovid. *Commun. Chem.* **2022**, *5*, 156.
5. Karmakar, A.; Basha, M.; Venkatesh Babu, G. T.; Botlagunta, M.; Malik, N. A.; Rampulla, R.; Mathur, A.; Gupta, A. K. Tertiary-Butoxycarbonyl (Boc) – A Strategic Group for *N*-Protection/Deprotection in the Synthesis of Various Natural/Unnatural *N*-Unprotected Aminoacid Cyanomethyl Esters. *Tetrahedron Lett.* **2018**, *59*, 4267–4271.
6. Lipshutz, B. H.; Ghorai, S.; Abela, A. R.; Moser, R.; Nishikata, T.; Duplais, C.; Krasovskiy, A.; Gaston, R. D.; Gadwood, R. C. TPGS-750-M: A Second-Generation Amphiphile for Metal-Catalyzed Cross-Couplings in Water at Room Temperature. *J. Org. Chem.* **2011**, *76*, 4379-4391.

7. Experimental Data

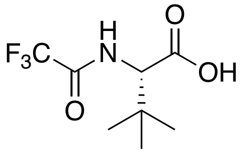

(*S*)-3,3-Dimethyl-2-(2,2,2-trifluoroacetamido)butanoic acid (1): ^{1}H NMR (400 MHz, DMSO-d$_6$) δ 9.44 (d, *J* = 8.9 Hz, 1H), 4.21 (d, *J* = 8.8 Hz, 1H), 1.00 (s, 9H). **^{13}C NMR (126 MHz, DMSO)** δ 171.4, 157.2 (q, *J* = 37.0 Hz), 116.4 (q, *J* = 287.7 Hz), 73.7, 61.6, 61.5, 34.1, 27.0. **^{19}F NMR (471 MHz, DMSO-d$_6$)** δ -73.2. **R$_f$** = 0.21 (10% MeOH/CH_2Cl_2, bromocresol green stain). Spectral data matched those previously reported.[1]

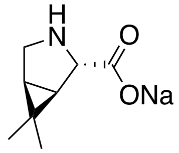

Sodium (1*R*,2*S*,5*S*)-6,6-dimethyl-3-azabicyclo[3.1.0]hexane-2-carboxylate (2): ^{1}H NMR (500 MHz, D_2O) δ 3.40 (s, 1H), 3.26 (dd, *J* = 11.2, 5.2 Hz, 1H), 2.80 (d, *J* = 11.2 Hz, 1H), 1.50 – 1.42 (m, 2H), 1.02 (d, *J* = 3.2 Hz, 3H), 1.00 (s, 3H). **^{13}C NMR (126 MHz, D_2O)** δ 182.6, 62.4, 45.7, 35.6, 30.1, 25.9, 19.4, 12.9. R$_f$ = N/A for sodium salt. **HRMS (**ESI, ToF**)** *m/z*: $[M+H]^+$ calcd. for $C_8H_{13}NO_2Na$ 178.0843; Found: 178.0806.

Note: The extra peaks observed in the NMR spectrum are due to the protonation of the sodium salt due to the acidic nature of D_2O resulting in the corresponding amino acid (see section 6).

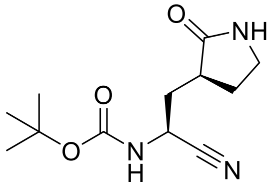

***t*-Butyl ((*S*)-1-cyano-2-((*S*)-2-oxopyrrolidin-3-yl)ethyl)carbamate (9): ^{1}H NMR (600 MHz, CD_3OD) δ** 4.72 (dd, *J* = 10.2, 8.2 Hz, 1H), 3.40 – 3.32 (m, 2H, partially obscured by solvent peak), 2.59-2.44 (m, 1H), 2.36 (dddd, *J* = 11.8, 8.6, 6.7, 3.0 Hz, 1H), 2.21 (ddd, *J* = 15.0, 9.3, 5.7 Hz, 1H), 1.92 – 1.80 (m, 2H), 1.46 (s, 9H). **^{13}C NMR (101 MHz, CD_3OD) δ** 181.1, 157.2, 120.5, 81.6, 41.9, 41.6, 39.3, 35.5, 28.7. **R$_f$:** 0.65 (10% MeOH/CH_2Cl_2, I_2 stain). Spectral data matched those previously reported.[2]

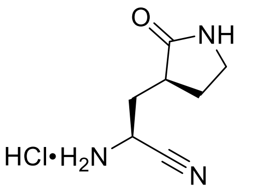

(*S*)-2-Amino-3-((*S*)-2-oxopyrrolidin-3-yl)propanenitrile hydrochloride (4): **^{1}H NMR (400 MHz, CD_3OD) δ** 4.81 (dd, apparent triplet, *J* = 7.3, 7.3 Hz, 1H), 3.45 – 3.35 (m, 2H), 2.78 (ddt, *J* = 10.8, 8.7, 7.3 Hz, 1H), 2.52 – 2.37 (m, 1H), 2.22 (t, *J* = 7.5 Hz, 2H), 1.91 (ddt, *J* = 12.6, 10.8, 9.2 Hz, 1H). **^{13}C NMR (101 MHz, CD_3OD) δ** 180.9, 117.2, 42.2, 41.9, 41.1, 34.2, 29.4. **R_f:** N/A for hydrochloride salt.

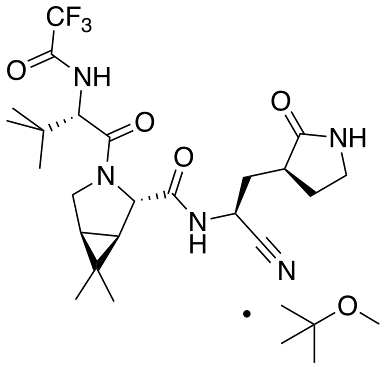

(1*R*,2*S*,5*S*)-*N*-((*S*)-1-Cyano-2-((*S*)-2-oxopyrrolidin-3-yl)ethyl)-3-((*S*)-3,3-dimethyl-2-(2,2,2-trifluoroacetamido)butanoyl)-6,6-dimethyl-3-azabicyclo[3.1.0]hexane-2-carboxamide MTBE solvate (Nirmatrelvir MTBE solvate; 6): ^{1}H NMR (500 MHz, DMSO-d_6) δ 9.40 (d, *J* = 8.5 Hz, 1H), 9.02 (d, *J* = 8.6 Hz, 1H), 7.67 (s, 1H), 4.97 (ddd, *J* = 10.5, 8.5, 5.1 Hz, 1H), 4.41 (d, *J* = 8.6 Hz, 1H), 4.16 (s, 1H), 3.91 (dd, *J* = 10.5, 5.5 Hz, 1H), 3.69 (d, *J* = 10.4 Hz, 1H), 3.14 (t, *J* = 9.2 Hz, 1H), 3.09 – 3.00 (m, 4H), 2.40 (qd, *J* = 10.2, 4.2 Hz, 1H), 2.19 – 2.04 (m, 2H), 1.71 (tq, *J* = 11.7, 7.2 Hz, 2H), 1.57 (dd, *J* = 7.7, 5.4 Hz, 1H), 1.32 (d, *J* = 7.5 Hz, 1H), 1.10 (d, *J* = 1.5 Hz, 9H), 1.03 (s, 3H), 0.98 (s, 9H), 0.85 (s, 3H). **^{13}C NMR (126 MHz, DMSO-d_6)** δ 177.5, 170.7, 167.5, 157.0 (q, *J* = 36.9 Hz), 119.6, 115.9 (q, *J* = 287.8 Hz), 72.1, 60.6, 60.1, 58.2, 48.7, 47.6, 37.8, 37.7, 36.8, 36.3, 34.6, 34.2, 33.2, 32.9, 30.3, 27.4, 26.9, 26.8, 26.3, 25.7, 19.3, 18.9, 13.0, 12.3. **^{19}F NMR (471 MHz, DMSO-d_6)** δ -72.9. Spectral data matched those previously reported.[1]

8. ^{1}H, ^{13}C, ^{19}F NMR Spectra of synthesized products

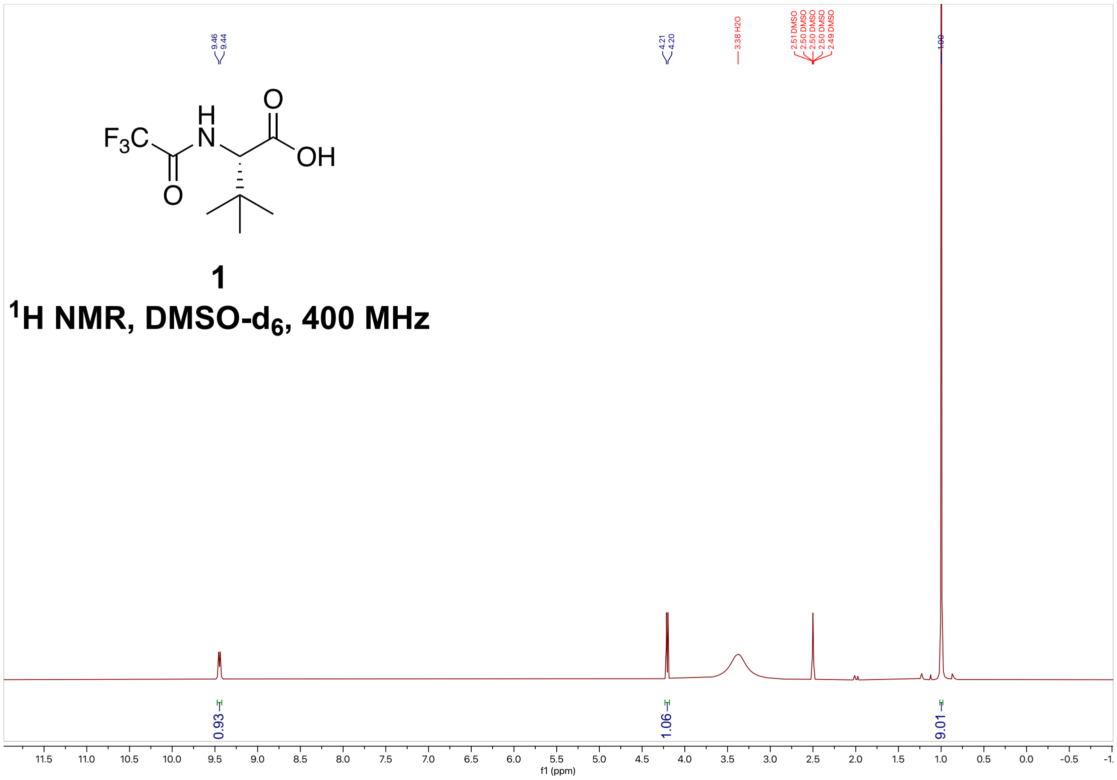
F3C
H
N
O
OH
O
1
1H NMR, DMSO-d6, 400 MHz
9.46
9.44
4.21
4.20
3.38 H2O
2.51 DMSO
2.50 DMSO
2.50 DMSO
2.50 DMSO
2.49 DMSO
1.00
0.93
1.06
9.01
f1 (ppm)

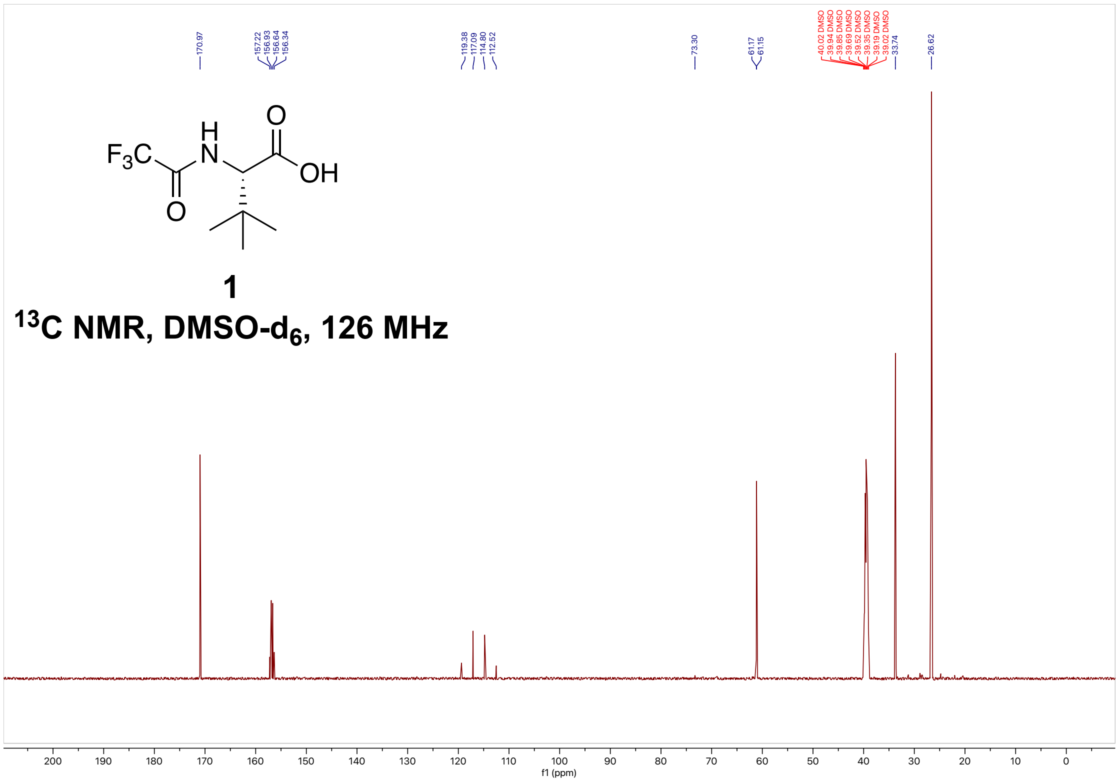
F3C
H
N
O
OH
O
1
13C NMR, DMSO-d6, 126 MHz
170.97
157.22
156.93
156.64
156.34
119.38
117.09
114.80
112.52
73.30
61.17
61.15
40.02 DMSO
39.94 DMSO
39.85 DMSO
39.69 DMSO
39.52 DMSO
39.35 DMSO
39.19 DMSO
39.02 DMSO
33.74
26.62
f1 (ppm)

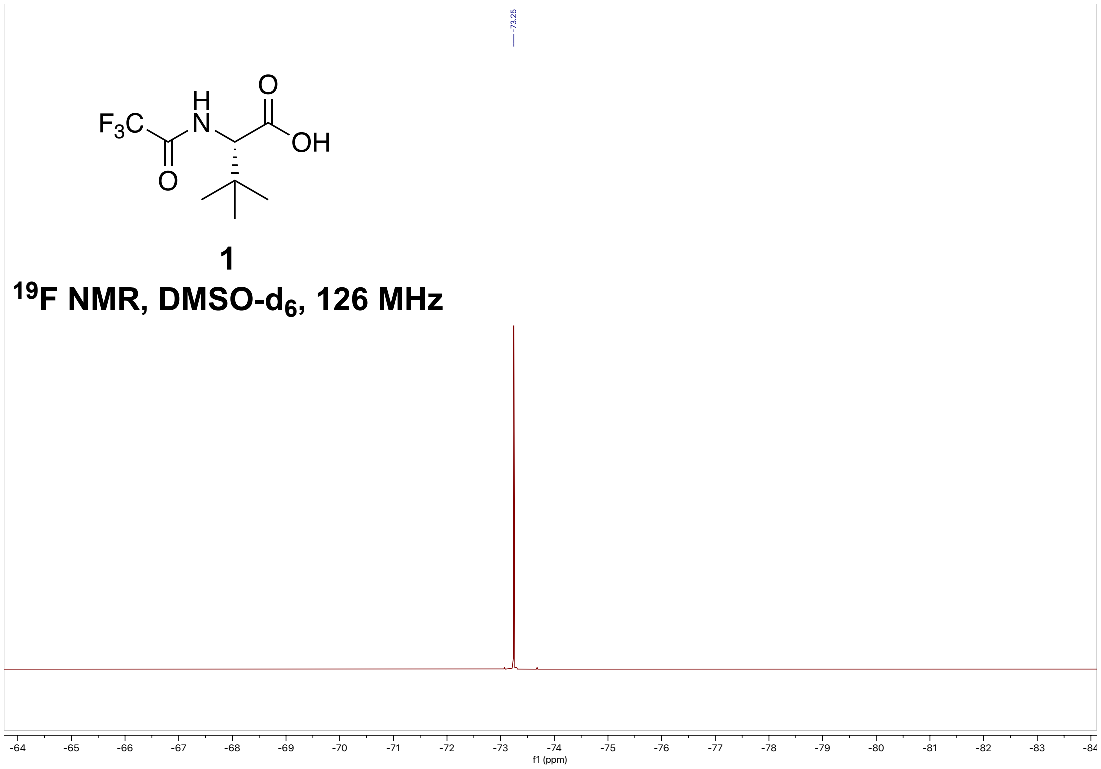

-73.25
F3C
H
N
O
OH
O
1
19F NMR, DMSO-d6, 126 MHz
-64 -65 -66 -67 -68 -69 -70 -71 -72 -73 -74 -75 -76 -77 -78 -79 -80 -81 -82 -83 -84
f1 (ppm)

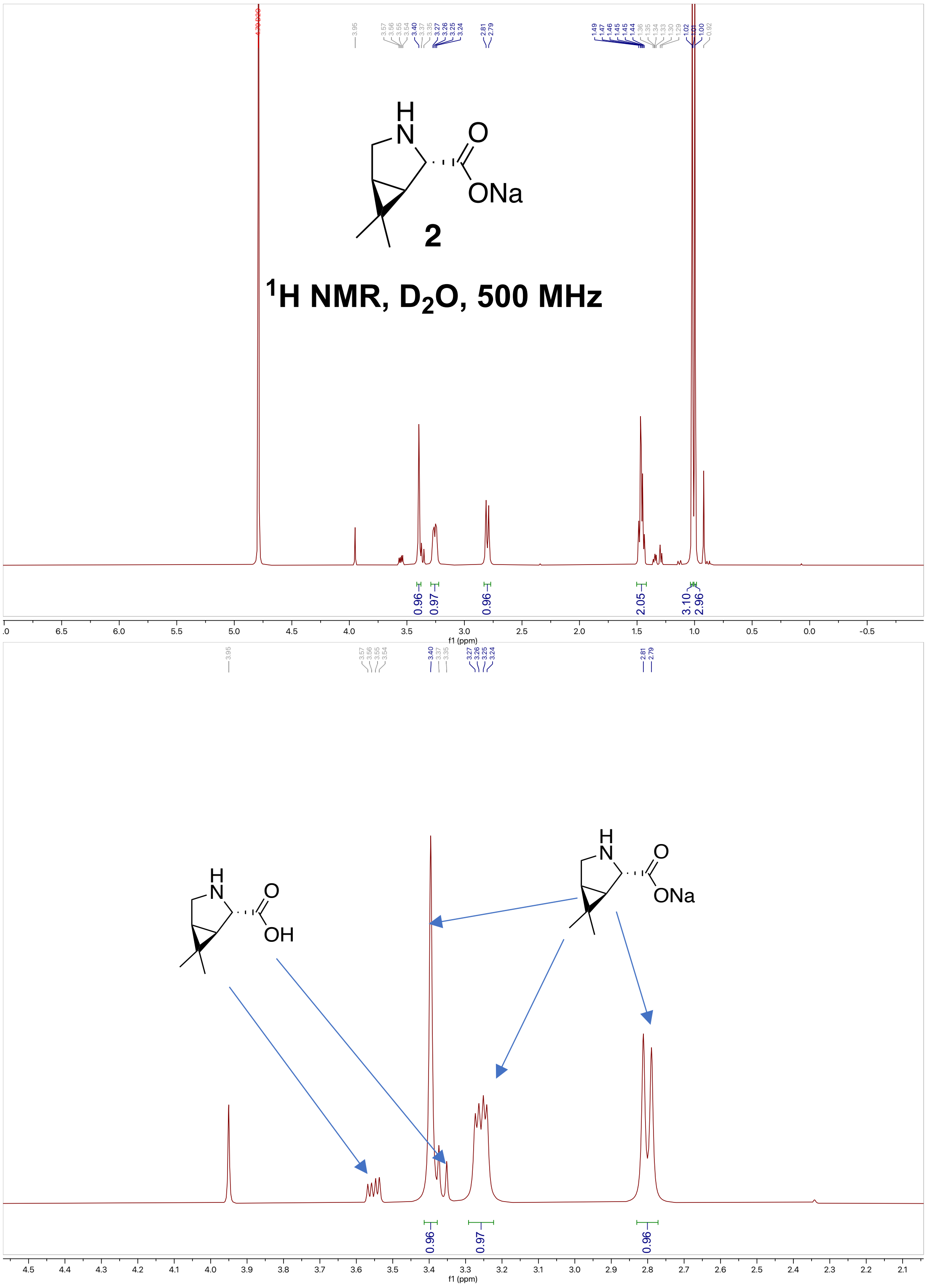

Figure S4: Blow-up of ^{1}H NMR spectrum of **2** to indicate protonation of the Na salt due to acidic nature of D_2O.

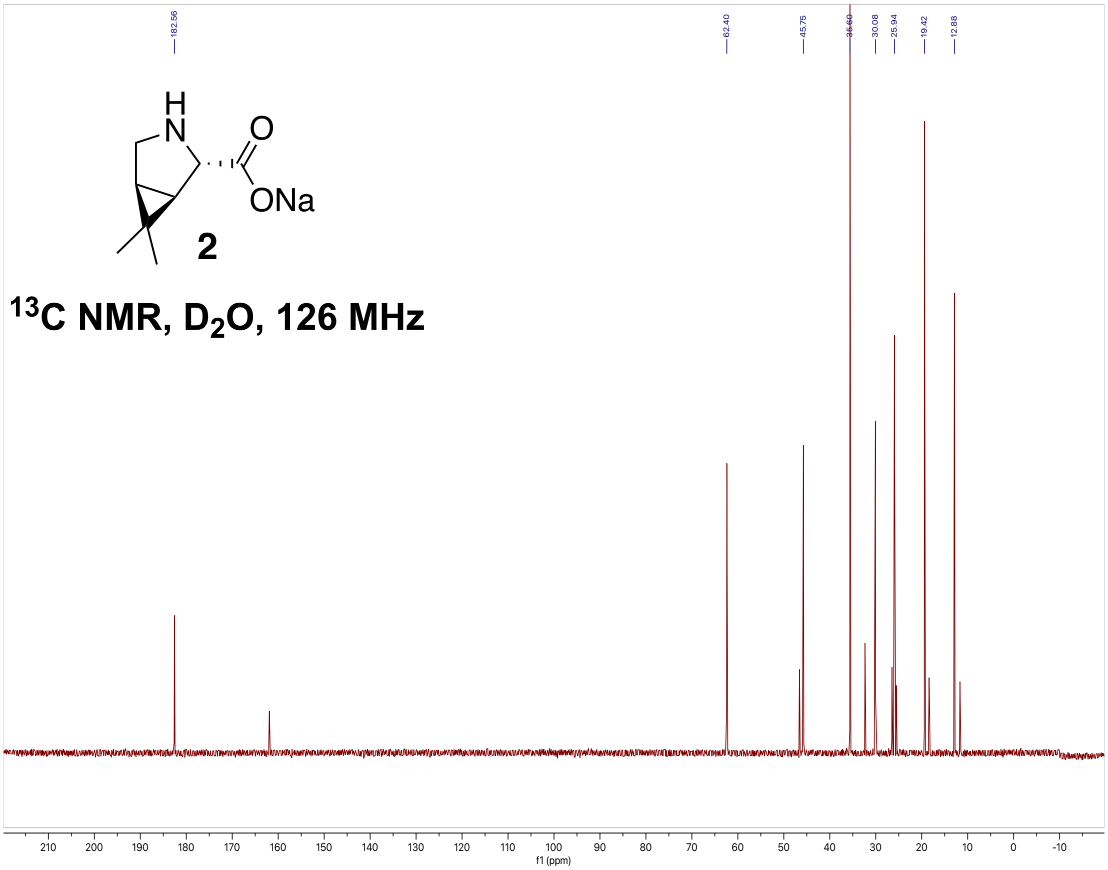
182.56
62.40
45.75
35.60
30.08
25.94
19.42
12.88
H
N
O
ONa
2
^{13}C NMR, D_2O, 126 MHz
210 200 190 180 170 160 150 140 130 120 110 100 90 80 70 60 50 40 30 20 10 0 -10
f1 (ppm)

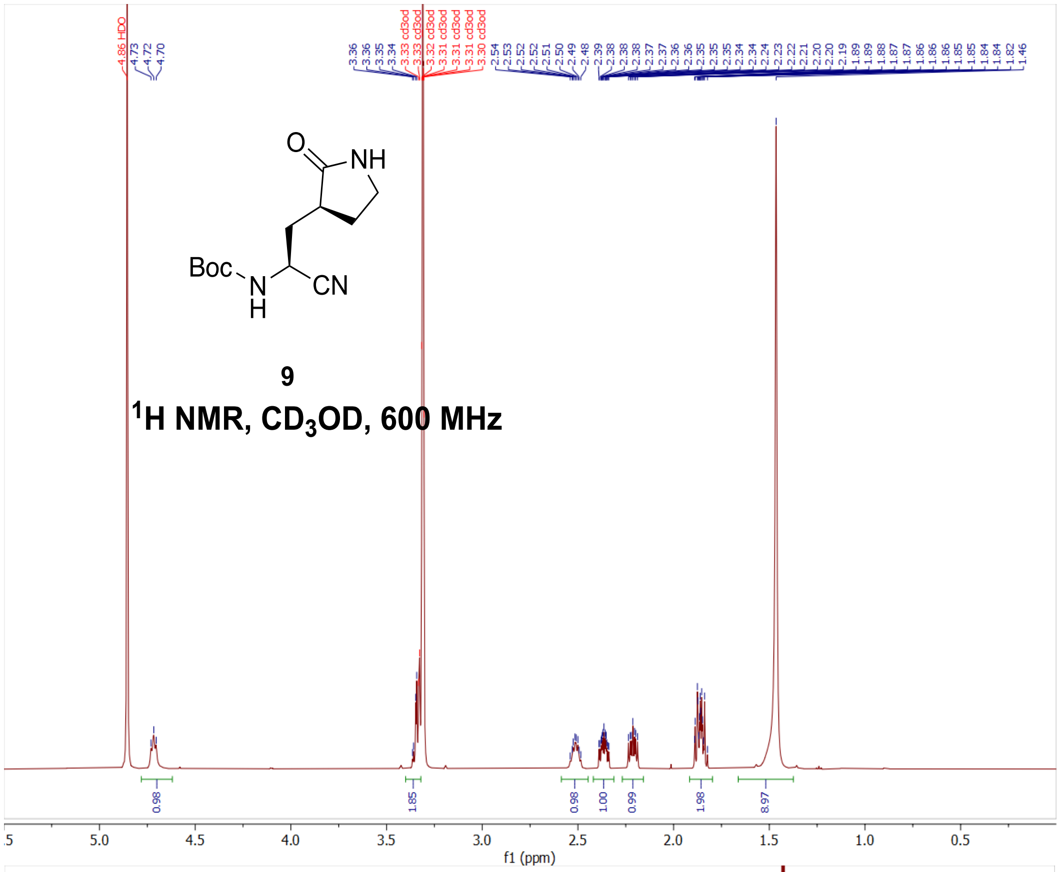
O
NH
Boc
N
H
CN
9
1H NMR, CD3OD, 600 MHz
f1 (ppm)

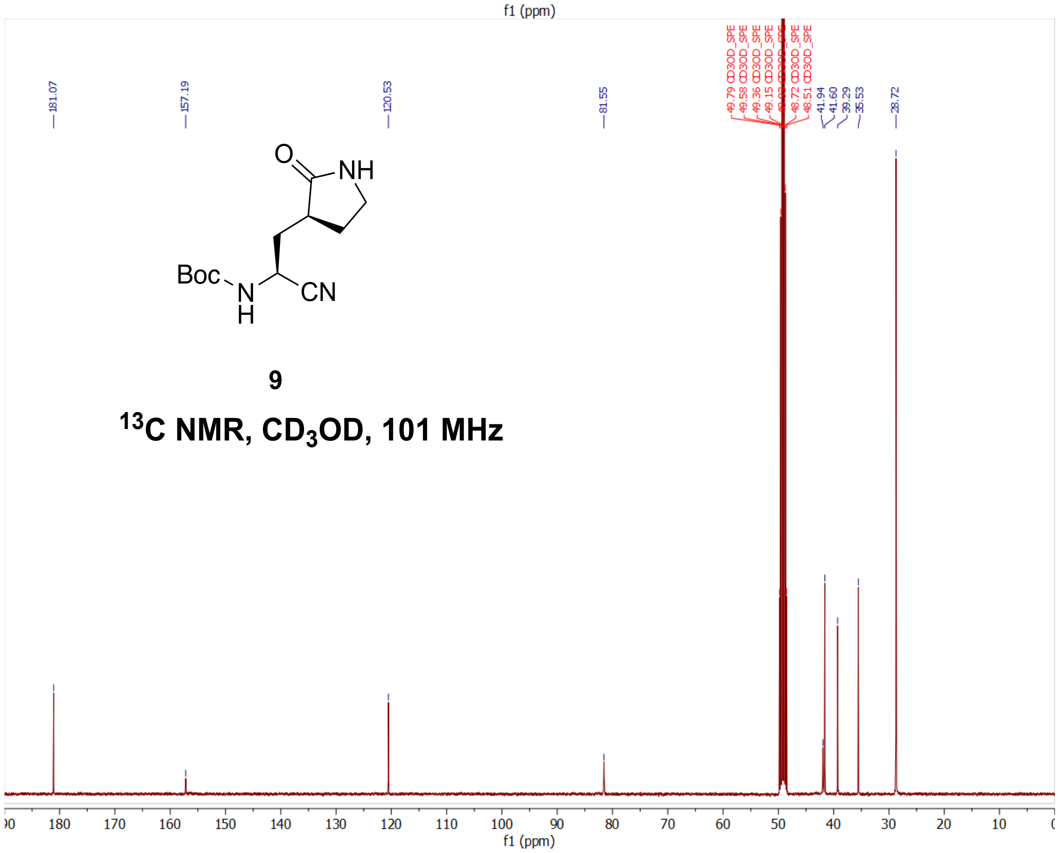
181.07
157.19
120.53
81.55
41.94
41.60
39.29
35.53
28.72
O
NH
Boc
N
H
CN
9
13C NMR, CD3OD, 101 MHz
f1 (ppm)

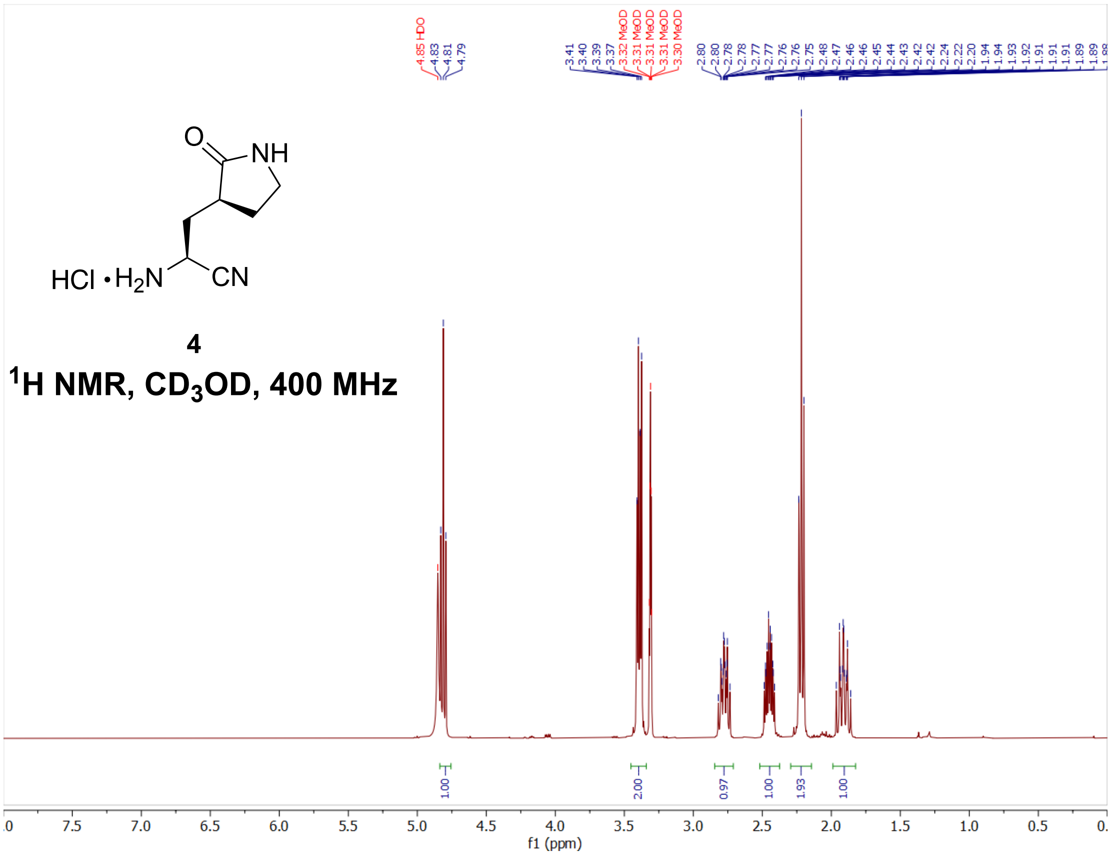
O
NH
HCl • H_2N
CN
4
^1H NMR, CD_3OD, 400 MHz
4.85 HDO
4.83
4.81
4.79
3.41
3.40
3.39
3.37
3.32 MeOD
3.31 MeOD
3.31 MeOD
3.31 MeOD
3.31 MeOD
3.30 MeOD
2.80
2.80
2.78
2.78
2.77
2.77
2.76
2.76
2.75
2.48
2.47
2.46
2.46
2.45
2.44
2.43
2.42
2.42
2.24
2.22
2.20
1.94
1.94
1.93
1.92
1.91
1.91
1.91
1.89
1.00
2.00
0.97
1.00
1.93
1.00
f1 (ppm)

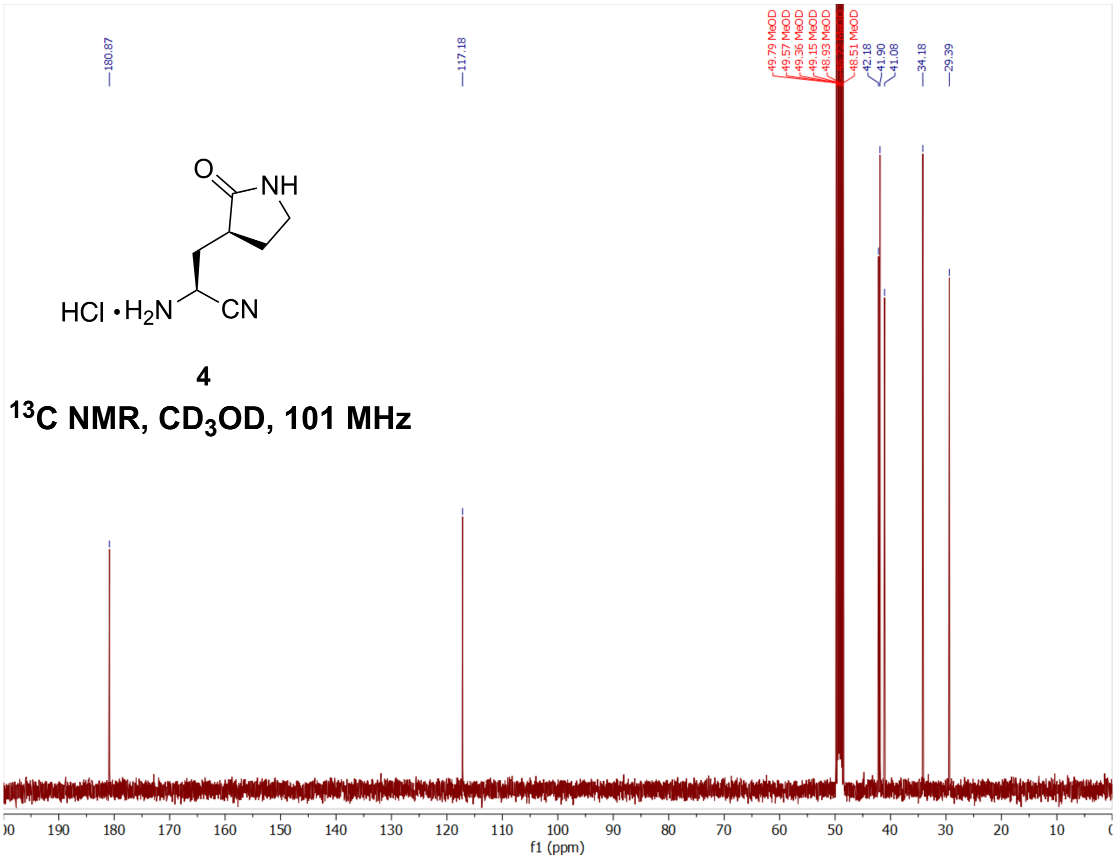
O
NH
HCl • H_2N
CN
4
^{13}C NMR, CD_3OD, 101 MHz
180.87
117.18
49.79 MeOD
49.57 MeOD
49.36 MeOD
49.15 MeOD
48.93 MeOD
48.51 MeOD
42.18
41.90
41.08
34.18
29.39
f1 (ppm)

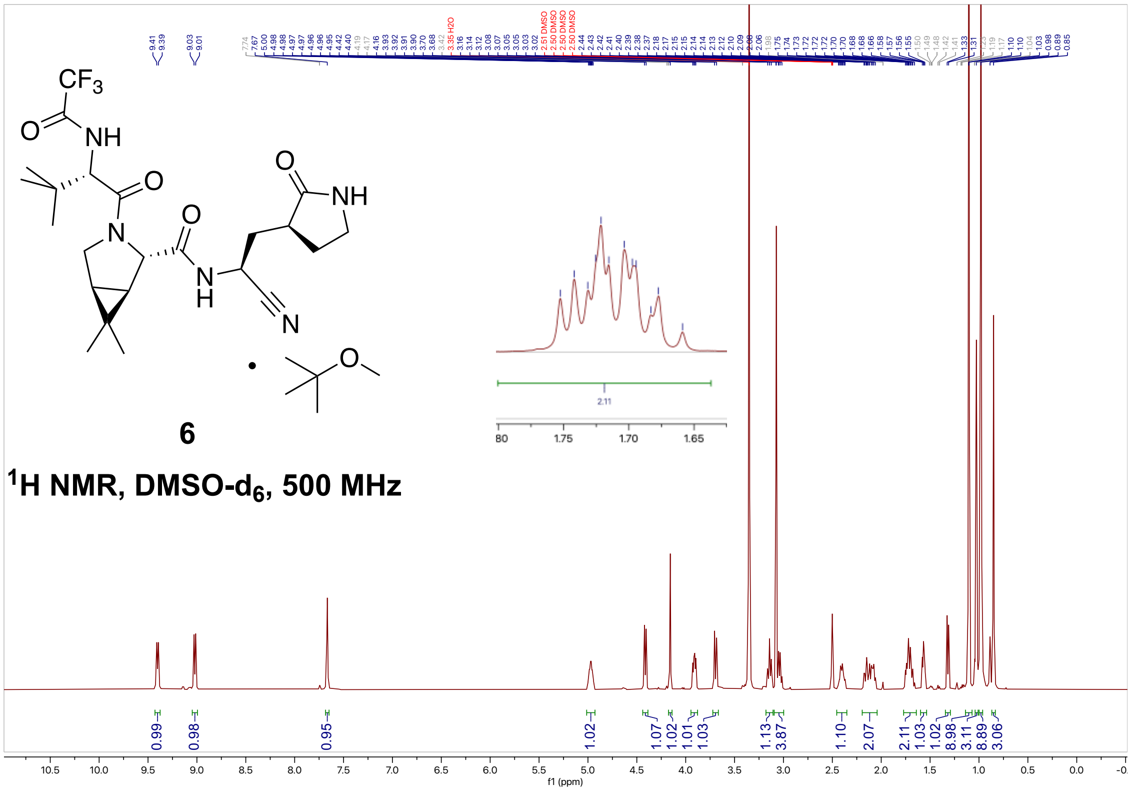
6
¹H NMR, DMSO-d6, 500 MHz
f1 (ppm)

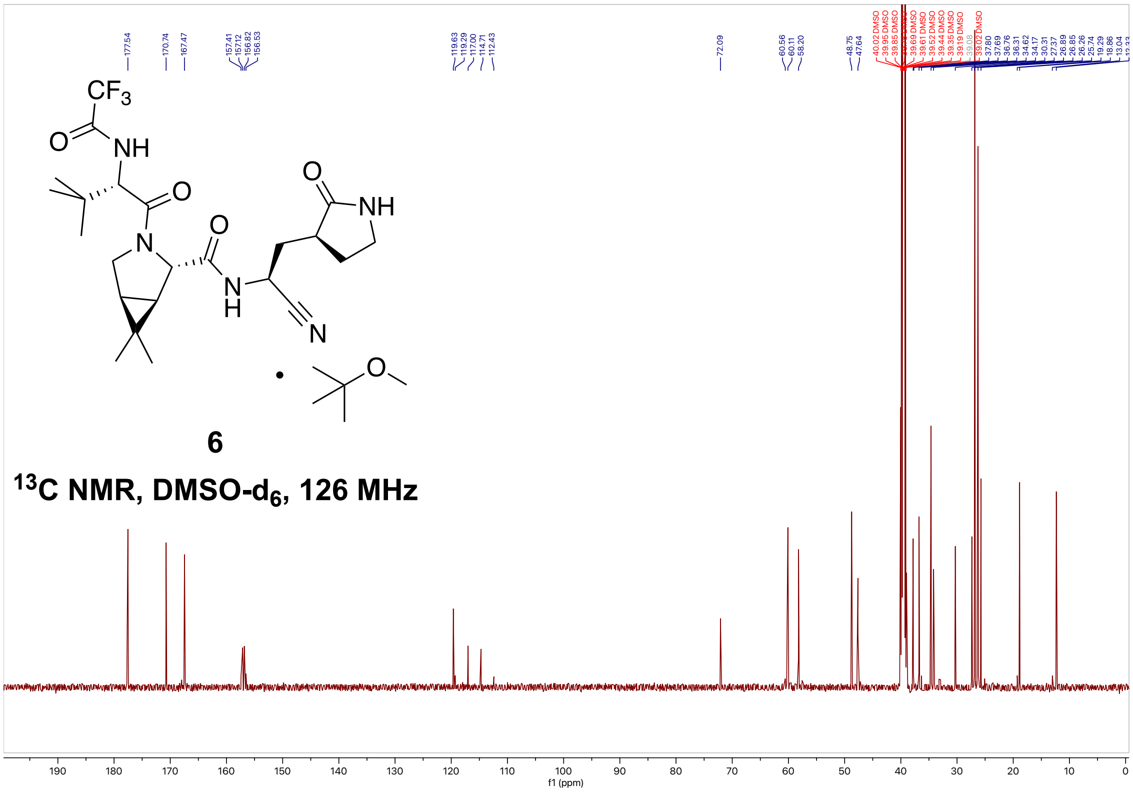
6
¹³C NMR, DMSO-d6, 126 MHz
f1 (ppm)

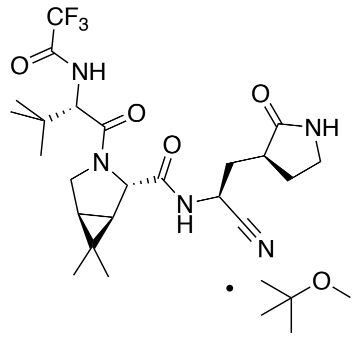

6

^{19}F NMR, DMSO-d$_6$, 471 MHz

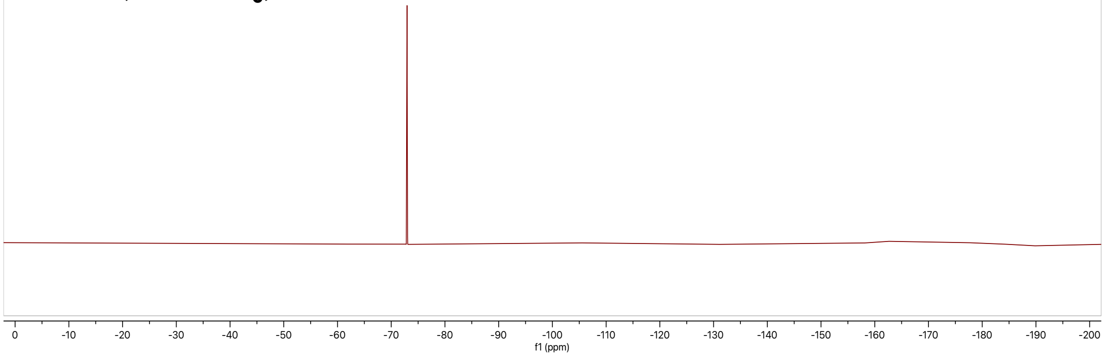

Supplement: Supplementary file 1 — ol2c03683_si_001.pdf [file ol2c03683_si_001.pdf]
